# A High-Throughput Search for SFXN1 Physical Partners Led to the Identification of ATAD3, HSD10 and TIM50

**DOI:** 10.3390/biology11091298

**Published:** 2022-08-31

**Authors:** Nesrine Tifoun, Mourad Bekhouche, José M. De las Heras, Arnaud Guillaume, Sylvina Bouleau, Isabelle Guénal, Bernard Mignotte, Nathalie Le Floch

**Affiliations:** 1 Laboratoire de Génétique et Biologie Cellulaire, UVSQ, Université Paris-Saclay, 78000 Versailles, France; 2 Ecole Pratique des Hautes Etudes, PSL Research University, 75014 Paris, France; 3 IUT de Vélizy/Rambouillet, UVSQ, Université Paris-Saclay, 78120 Rambouillet, France

**Keywords:** sideroflexin, SFXN1, mitochondria, ATAD3A, 17β-HSD10, TIM50

## Abstract

**Simple Summary:**

Mitochondria are central players in cell fate and cell death. Indeed, mitochondrial dysfunction has been observed in many diseases, including neurodegenerative diseases. The activity of these organelles relies on numerous mitochondrial transporters, among which the sideroflexins have received little attention to date despite their emerging importance in human health. To better understand the cellular functions of these transporters and their associations with diseases, we herein investigated the molecular partners of one human sideroflexin, SFXN1. Several proteins capable of interacting with SFXN1 were identified, including ATAD3 and HSD10, two mitochondrial proteins linked to neuronal disorders.

**Abstract:**

Sideroflexins (SFXN, SLC56) are a family of evolutionarily conserved mitochondrial carriers potentially involved in iron homeostasis. One member of the SFXN family is SFXN1, recently identified as a human mitochondrial serine transporter. However, little is known about the SFXN1 interactome, necessitating a high-throughput search to better characterize SFXN1 mitochondrial functions. Via co-immunoprecipitation followed by shotgun mass spectrometry (coIP-MS), we identified 96 putative SFXN1 interactors in the MCF7 human cell line. Our in silico analysis of the SFXN1 interactome highlights biological processes linked to mitochondrial organization, electron transport chains and transmembrane transport. Among the potential physical partners, ATAD3A and 17β-HSD10, two proteins associated with neurological disorders, were confirmed using different human cell lines. Nevertheless, further work will be needed to investigate the significance of these interactions.

## 1. Introduction

Sideroflexins (SFXN/SLC56) are a family of poorly characterized mitochondrial solute carriers with five members in humans and rodents (SFXN1-SFXN5), two members in *Drosophila* (dSfxn1/3 and dSfxn2) and only one in *S. cerevisiae* (Fungal SideroFlexin 1, FSF1) [1,2,3]. The high degree of conservation among eukaryotic SFXN suggests that these proteins ensure major mitochondrial functions. Accordingly, loss-of-function mutations in *SFXN4* were described in a rare mitochondrial disease associated with oxidative phosphorylation (OXPHOS) deficiency [4,5,6]. Downregulations of SFXN1 and SFXN3 in Alzheimer’s (AD) and Parkinson’s (PD) diseases were also reported [7,8]. SFXN3, the closest homologue of SFXN1, is enriched in neurons, may participate in synaptogenesis and the maintenance of synapse morphology and has been associated to PD [9,10]. In *Drosophila*, dSfxn1/3 overexpression counteracts the loss of dopaminergic neurons in a PD model, suggesting a neuroprotective role of SFXN [11]. Furthermore, SFXN3 mutations lead to retinal degeneration in mice [12].

For two decades, SFXN were presumed to be metabolite transporters on the basis of in silico structural analysis. For example, S. cerevisiae Fsf1 (YOR271cp) was proposed to be an alpha-isopropylmalate carrier though no experimental data has ascertained this function [13]. Since a mitochondrial iron overload was seen in flexed-tail mice carrying a mutation in the SFXN1 gene, SFXN1 was suggested as functioning in iron homeostasis. It was thus postulated that SFXN proteins mediated the mitochondrial uptake of a metabolite required for iron metabolism [14]. Indeed, increased mitochondrial iron levels and deficient heme biosynthesis were reported in SFXN2 and SFXN4 KO human cells [15,16]. However, the molecular mechanisms that underlie this imbalance in iron homeostasis remain unknown. Recently, via a CRISPR-based screen in human leukemic cells, SFXN1 was identified as a mitochondrial serine transporter, thus connecting SFXN1 with the one carbon metabolism (OCM), an essential pathway supporting physiological processes such as the synthesis of purines and amino acid homeostasis [17,18]. The role of SFXN1 in the OCM pathway has provided clues for the regulation of heme biosynthesis by sideroflexins [17]. Specifically, heme biosynthesis relies on the availability of the precursor glycine, which itself is provided by the catabolism of serine. Subsequently, two studies confirmed the relationships between SFXN1 and heme metabolism: SFXN1 loss-of-function in mammalian cells and zebrafish leads to impaired heme biosynthesis [19,20]. Collectively, these data have shed light on the role of the SFXN family in regulating essential metabolic pathways and mitochondrial activities. Despite the recent progress, still little is known about the molecular functions of the SFXN family.

The knowledge of the SFXN interactome may assist in elucidating the functions of these transporters, specifically the mechanisms by which they regulate mitochondrial activities. The main objective of our study was to uncover the physical partners of the human SFXN1 because (i) SFXN1 is the most conserved member of the SFXN family, and (ii) it appears as a main regulator of mitochondrial function through its involvement in OCM. Upon large-scale survey of the SFXN1 interactome, via coIP-MS/MS on mitochondria of MCF7 human cells, we identified two proteins related to neurodegeneration, namely ATAD3 and the multifunctional enzyme 17β-hydroxysteroid dehydrogenase (17β-HSD10). Taken together, these data may help with better understanding the role of SFXN1 in pathophysiology, notably in neurological disorders.

## 2. Materials and Methods

### 2.1. Cell Lines and Cell Culture

HEK-293T, HeLa, HepG2, HT1080, MCF7, MDA-MB-231, MDA-MB-468, MEFSV40, PC12, RKO and SH-SY5Y cells were grown in DMEM medium (Thermo Fisher, Waltham, MA, USA). KGN, COV434 and HCT116 were grown in DMEM/F12 medium (Thermo Fisher, Waltham, MA, USA). A2780, A2780Cis, and T47D cells were grown in RPMI 1640 medium (Thermo Fisher, Waltham, MA, USA). All media were supplemented with 10% FBS (Thermo Fisher), 1% GlutaMAX (Thermo Fisher), 100 μg/mL penicillin and 100 U/mL streptomycin (Thermo Fisher). Cells were cultured as monolayers at 37 °C in a humidified atmosphere with 5% (*v*/*v*) CO_2_ and were maintained in culture for no more than 3 months (less than 30 passages).

### 2.2. Plasmids and siRNA Transient Transfections

Plasmids encoding FLAG-SFXNs were obtained from GenScript. Empty vector (pcDNA3.1D V5-His) was obtained from Invitrogen. HEK293T cells were seeded into 60 mm Petri dishes (1 × 10^6^ cells/dish) and transfected with 3 μg of DNA using Lipofectamine LTX (Life Technologies, Carlsbad, CA, USA) following manufacturer instructions. For RNA interference (RNAi) experiments, MCF7 cells were transiently transfected with either a scrambled control siRNA (Control siRNA-A sc-37007, Santa Cruz Biotechnology, Inc., Heidelberg, Germany) or a pool of specific siRNA for SFXN1 (sc-91814, Santa Cruz Biotechnology, Inc., Heidelberg, Germany). Transfection was performed using Interferin™ transfection reagent following manufacturer instructions (Polyplus-Transfection Inc., New York, NY, USA). Briefly, a mix of siRNA and Interferin™ transfection reagent was incubated for 10 min at room temperature (RT) and added to each well at a final concentration of 10 nM. Cells were incubated at 37 °C under standard culture conditions and collected after 3, 4, or 7 days to extract proteins.

### 2.3. Protein Extraction and Subcellular Fractionation

For total protein extraction, 2 × 10^6^ cells were grown in 60 mm dishes, trypsinized 24 h post-seeding, harvested and centrifuged at 200× *g* for 5 min. Cell pellets were homogenized in lysis buffer (250 mM NaCl, 50 mM HEPES, 5 mM EDTA, 0.1% NP40, 0.1 M DTT) supplemented with 1/100 protease inhibitors cocktail (Cat. No. 04693116001, Roche, Mannheim, Germany) and 0.2 mM sodium orthovanadate/phosphatase inhibitors (cat.567540, Calbiochem, San Diego, CA, USA).

### 2.4. Mitochondria Enrichment from Mammalian Cells and Drosophila Larvae

For mitochondrial enrichment from human cells, 1.5 × 10^7^ cells grown on 100 mm dishes were harvested, centrifuged and washed with PBS. Subcellular fractionation by a differential centrifugation was performed as previously described [21]. Briefly, cells were harvested, washed in PBS and centrifuged at 200× *g* for 5 min. Cell pellets were resuspended in fractionation buffer A (250 mM sucrose, 0.1 mM EDTA, 1 mM EGTA, 10 mM Hepes-KOH pH 7.4) and incubated for 30 min at 4 °C. Cell disruption was performed by passing the cells through a 26-gauge needle 15 times. The homogenates were centrifuged at 700× *g* for 15 min at 4 °C to pellet cell debris, nuclei and intact cells. The supernatants were collected and further centrifuged at 7000× *g* for 20 min at 4 °C to pellet mitochondria. The supernatant was designated as cytosolic fraction. The mitochondria pellets were washed in fractionation buffer B (250 mM sucrose, 5 mM succinate, 5 mM KH_2_PO_4_, 10 mM Hepes-KOH pH 7.4) and centrifuged at 7000× *g* for 20 min at 4 °C. Purified mitochondria were lysed in TBS-CHAPS 2% (EUROMEDEX, Strasbourg, France) supplemented with protease inhibitors. All buffers were supplemented with 1 mM protease inhibitors (Roche, Mannheim, Germany). Protein concentration of cell extracts and fractions were determined via Bradford assay.

*Drosophila* larvae were dissected in PBS pH 7.6, crushed with a mechanical pestle in 400 μL of fractionation buffer (Tris-HCl 10 mM pH 8, EDTA 10 mM, sucrose 0.32 M), supplemented with a protease inhibitor cocktail (Mini complete without EDTA, Roche). Debris were removed by centrifugation for 5 min at 300× *g* at 4 °C. A sample of supernatant (total extracts) was kept for further analysis. An additional centrifugation was carried out at 2650× *g* for 10 min at 4 °C. The supernatant was recovered (cytosolic fraction). The pellet, i.e., mitochondrial fraction, was washed once with fractionation buffer, centrifuged for 10 min at 2650× *g* at 4 °C and resuspended in 300 μL of fractionation buffer. Samples were denatured with Laemmli buffer 1×, DTT 0.1 M and boiled for 5 min at 96 °C and stored at −20 °C before Western blot analysis.

### 2.5. Western Blot

Equal quantities of proteins (10 to 30 μg) were loaded on Mini-PROTEAN TGX Stain Free precast polyacrylamide 4–20% gels (BIORAD, Hercules, CA, USA) in 1× Tris Glycine-SDS buffer. Proteins were transferred onto Immobilon-P PVDF membranes (Millipore, Darmstadt, Germany) in 1× Tris Glycine with 20% ethanol. Stain-free technology enables the visualization of total proteins adsorbed onto the membrane without the use of any dye. For immunoblotting, antibodies were diluted in TBS-Tween 0.1%. The primary antibodies used for immunoblotting are listed in Table S54. HRP-coupled secondary antibodies were obtained from Jackson Immunoresearch and Diagomics. Chemiluminescent detection was performed with Clarity Western ECL substrate (BIORAD) and the signal was captured by Chemidoc (BIORAD). Quantification was performed using ImageLab software (BIORAD).

### 2.6. Co-Immunoprecipitation

Cells grown in a 100 mm dish were washed thrice in cold PBS. Then, cells were lysed with 1.2 mL NP40 lysis buffer (20 mM Tris, pH 8, 150 mM NaCl, 0.5% NP-40, 0.5% sodium deoxycholate containing a protease inhibitor cocktail (Cat. No. 04693116001, Roche, Mannheim, Germany). The lysates were centrifuged at 200× *g* for 5 min. The supernatant was transferred to a new tube and precipitated with 2 μg rabbit anti-SFXN1 antibody or rabbit anti-IgG antibody for negative control. The immune complexes formed were then incubated for 30 min (1000 rpm, RT) with G protein-coupled magnetic beads (Bio-Adembeads PAG 0463, Ademtech, Pessac, France) previously equilibrated with lysis buffer. The supernatant was collected to ensure the depletion of the immunoprecipitated protein, and the pellet was washed 3 times with lysis buffer to remove non-specific binding. For elution, beads were then resuspended with 30 μL of elution buffer (50 mM Tris-HCl pH 8.0, 10 mM EDTA pH 8.0, 1% SDS) for 4 min at 37 °C.

### 2.7. Immunofluorescence Staining

1 × 10^6^ cells were plated on 60 mm-diameter Petri dishes containing coverslips and fixed 24 h post-seeding, at a confluence of 60%, with 3.7% cold paraformaldehyde (PFA) for 20 min. Cells were washed once with PBS and then permeabilized with 0.5% Tween20 for 30 min. Nonspecific sites were blocked with 1% PBS-BSA for 30 min. immunostaining, and then cells were incubated for 60 min with primary or secondary antibodies in 3% PBS-BSA. Coverslips were mounted with ProLong Gold Antifade Mountant (Invitrogen). Imaging was performed using Leica TCS SPE or Leica TCS SP8 confocal microscopes (CYMAGES imaging facility, UVSQ). Image analysis was performed using ImageJ software.

### 2.8. Imaging and Statistical Analysis for Co-Localization

Images were taken under a Leica SP8-X confocal microscope using a x63 oil immersion objective with 1.4 NA, 1024 × 1024 resolution and Voxel size x = 0.18, y = 0.18, z = 0.3.

The cell of interest was cropped and properly denoised (background subtracted and filtered with a Gaussian blur filter), and individual pixel intensity in three consecutive stacks was extracted with Fiji package [22]. Data were processed, and graphs were generated in Microsoft Excel (2019), R package (R Core Team, 2020) and GraphPad Prism 8.0. Co-occurrence (Manders’s overlap coefficient [23]) and correlation (Pearson’s correlation coefficient or r) values were calculated as reviewed in [24].

### 2.9. Sample Preparation for Proteomic Analyses

Enriched mitochondrial fraction from MCF-7 cells were prepared as described above. Protein content was estimated by Bradford assay. Proteins (250 μg per condition) were subjected to immunoprecipitation using magnetic beads (Bio-Adembeads, PAG 0463, Ademtech, Pessac, France). Briefly, 250 μg of enriched mitochondrial fraction was incubated in lysis buffer (20 mM Tris pH 8.0, 150 mM NaCl, 0.5% NP40, 0.5% deoxycholate complemented with anti-protease cocktail) supplemented with 2 μg of a rabbit anti-SFXN1 antibody (HPA063745, Sigma Aldrich, Saint-Quentin-Fallavier, France) or a rabbit IgG control antibody (Cat#12-370, Merck Millipore, Overijse, Belgium) for 30 min at RT at 1000 rpm on a tube rotator. After washing and equilibration in lysis buffer, 20 μL of magnetic beads were added to the immune complex and incubated 30 min under agitation (1000 rpm at RT). The magnetic beads were washed twice in lysis buffer, and the bound proteins were then eluted twice using 15 μL of PAG elution buffer (Cat#10701, Ademtech, Pessac, France). The acidic pH was neutralized with 1 volume (30 μL) of 20 mM Tris pH 8.0, 150 mM NaCl and 10 μL of 0.5 M Tris pH 7.5 before sample preparation for MS/MS injection.

The proteins extracted from eluates were incubated with 25 mM NH_4_HCO_3_ buffer containing sequencing-grade trypsin (0.4 μg for 55 μL; Promega, Charbonnières-les-Bains, France) overnight at 37 °C. Peptides were desalted using ZipTip μ-C18 Pipette Tips (Millipore). Samples were analyzed using an Orbitrap Q-Exactive Plus coupled to a Nano-LC Proxeon 1000 equipped with an easy spray ion source (Thermo Scientific, Waltham, MA, USA). On the Q-Exactive Plus instrument, peptides were loaded with an online preconcentration method, separated by chromatography using a Pepmap-RSLC C18 column (0.75 × 500 mm, 2 μm, 100 Å) from Thermo Scientific, equilibrated at 50 °C and operated at a flow rate of 300 nL/min. Peptides were eluted by a gradient of solvent A (H_2_O, 0.1% FA) and solvent B (100% ACN, 0.1% FA), the column was first equilibrated 5 min with 95% of A, then B was raised to 35% in 93 min and finally, the column was washed with 80% B during 10 min and re-equilibrated at 95% A over 10 min. Peptides were analyzed in the Orbitrap cell at a resolution of 70,000, with a mass range of *m*/*z* 375–1500 and an AGC target of 3 × 10^6^. Fragments were obtained by higher-energy collisional dissociation (HCD) activation with a collisional energy of 28% and a dynamic exclusion of 30 s. MS/MS data were acquired in the Orbitrap cell in a Top20 DDA mode, at a resolution of 17,500, with an AGC target of 2 × 10^5^. Monocharged peptides and unassigned charge states were excluded from the MS/MS acquisition. The maximum ion accumulation times were set to 50 ms for MS and 45 ms for MS/MS acquisitions (Proteomics/Mass Spectrometry Core Facility, Institut Jacques Monod, Paris, France).

### 2.10. Proteomic Data Analysis

All MS and MS/MS data were processed with the Proteome Discoverer software (Thermo Scientific, version 2.2) coupled to the Mascot search engine (Matrix Science, version 2.5.1). The mass tolerance was set to 6 ppm for precursor ions and 0.02 Da for fragments. The maximum number of missed cleavages was limited to two for the trypsin protease. The following variable modifications were allowed: oxidation (Met), phosphorylation (Ser, Thr or Tyr) and acetylation (protein N-terminus). The SwissProt database (2017_12 release) with the *Homo sapiens* taxonomy was used for the MS/MS identification step. Peptide identifications were validated using a 1% FDR (false discovery rate) threshold calculated with the Percolator algorithm. Proteins for which validated peptides were identified solely in the SFXN1 immunoprecipitation and not in the control were considered potential SFXN1 partners. The mass spectrometry proteomics data have been deposited to the ProteomeXchange Consortium via the PRIDE [25] partner repository with the dataset identifier PXD030749.

### 2.11. Biological Process Analysis Using PANTHER

Protein localization was determined using the gene ontology annotation [26,27]. Potential SFXN1 partners were submitted to the online PANTHER classification system using the PANTHER Overrepresentation test (released 20171205) with the whole annotation for *Homo sapiens* in the Gene Ontology database as reference set [28]. The overrepresented biological processes with a FDR < 0.05 were assumed to be related to the biological role of the SFXN1. Proteins that may not interact with SFXN1 were also submitted to the same analysis pipeline to further evaluate the specificity of the biological process determined.

### 2.12. Analysis of SFXN1 Interactome Using STRING

STRING version 11.0 [29] was used to model SFXN1 interactome with the following basic settings: experiments, database, co-expression and co-occurrence were chosen for active sources; the value for the minimum required interaction score was set at high confidence (0.700), and no more than 5 interactions for the first shell and none for the second shell were parameters chosen for the max number of interactors to show. The network generated can be retrieved at the following permalink: https://version-11-0.string-db.org/cgi/network.pl?networkId=jUrnsUFnafB2 (accessed on 1 november 2019). Functional enrichments for GO terms linked to cellular component permitted to retrieve mitochondrial proteins (GO:0031966), membrane mitochondrial proteins (GO:0005739) and inner membrane mitochondrial proteins (GO:0005743). A Venn diagram was drawn using the web tool accessible here: http://bioinformatics.psb.ugent.be/webtools/Venn/ (accessed on 20 december 2021).

### 2.13. Metascape Analysis of SFXN1 Physical Partners

A list containing the Uniprot identifiers for the 96 proteins found in our co-IP-MS/MS experiment was submitted to the Metascape webtool (https://metascape.org/gp/index.html#/main/step1 (accessed on 6 December 2021)). Express Analysis was chosen for enrichment and clustering analysis. Metascape Express analysis consists of an automated analysis workflow beginning with identifier conversion and followed by gene annotation, membership search and enrichment analysis. For the meta-analysis conducted to compare enriched terms related to mitochondrial proteins and SFXN1 partners, we submitted a list of 1484 proteins selected from the IMPI database (IMPI-2021-Q4pre) containing 1357 genes encoding verified mitochondrial proteins with “gold standard” evidence of mitochondrial localization and 127 encoding mitochondrial associated proteins with evidence of mitochondrial localization but lacking visual confirmation (https://www.mrc-mbu.cam.ac.uk/research-resources-and-facilities/impi (accessed on 27 July 2022)).

To construct the protein–protein interaction network for our 96 candidates, Metascape “Express Analysis” mode was chosen. It utilizes the “Physical Core” dataset, which contains only 2/3 of the highest-scoring corresponding STRING data. The MCODE algorithm automatically extracts densely connected protein complexes from our list of SFXN1 partners.

### 2.14. Oxygen Consumption Rate Measurement

Respiration was assessed in live cells using the Seahorse XF HS mini Analyzer (Agilent Technologies). The oxygen consumption rate (OCR) was determined with the Seahorse XF Cell Mito Stress Test Kit (Agilent Technologies #103015) according to the manufacturer’s instructions. Before the assay, 4 × 10^5^ of MCF7 cells were transfected in suspension in a 6-well plate with a scrambled siRNA (Control siRNA-A: sc-37007, Santa Cruz Biotechnology) or a pool of three SFXN1 siRNA (SFXN1 siRNA (h): sc-91814, Santa Cruz Biotechnology). Cells were trypsinized 48 h post-transfection and 2.5 × 10^5^ cells/well were plated on a polylysine-coated Seahorse XFp cell culture miniplate in Seahorse XF DMEM supplemented with 1 mM pyruvate, 2 mM glutamine and 10 mM glucose. Following a 1-h incubation at 37 °C without CO_2_, the plate was loaded into the Seahorse XF HS mini Analyzer, and the OCR was measured during the sequential addition of 1.5 μM oligomycin, 1 μM carbonylcyanide m-chlorophenylhydrazone (FCCP) and 0.5 μM antimycin A plus rotenone.

### 2.15. Proximity Ligation Assay

Proximity ligation assay experiment was done with the Duolink^®^ In Situ Red Starter Kit according to the manufacturer instructions (DUO92101, Sigma Aldrich, Saint-Quentin-Fallavier, France) on MCF-7 cells and using mouse anti-HSD10 antibody (sc-136326, Santa Cruz Biotechnology, Dallas, TX, USA) and rabbit anti-SFXN1 antibody (HPA063745, Sigma Aldrich, Saint-Quentin-Fallavier, France). A negative control without primary antibodies was also carried out. Briefly, MCF-7 cells were cultured on coverslips in 6-well plates. Cells were washed 3 times with PBS, fixed 10 min in 3.7% PFA and permeabilized 10 min with 0.1% Triton X-100 at 37 °C. The cells were washed thrice for 5 min with PBS between each step. The coverslips were placed in a humidified chamber and blocked with 50 μL Duolink blocking solution for 60 min at 37 °C. Primary antibodies at 2 μg/mL were incubated overnight at 4 °C. After 2 washes for 5 min with the provided wash buffer A, PLA probes were added and incubated for 1 h at 37 °C in a humidified chamber. After 2 washes of 5 min with wash buffer A, the ligase (diluted 1/40 in ligation buffer) was incubated 30 min at 37 °C. The coverslips were washed twice, each time for 5 min, with buffer A. Amplification step in the presence of the DNA polymerase was performed during 100 min at 37 °C in the dark. Coverslips were washed twice, each time 10 min with buffer B. Subsequently nuclei were stained with DAPI for 15 min and the cover-slips were mounted in ProLong Gold Antifade Mountant (Invitrogen). Slides were observed using a Leica TCS SPE confocal microscope with the DAPI and TRITC filters. The number of red dots was quantified using Fiji software and the Cell Counter plug-ins [22].

### 2.16. Biological Resources

The lists of antibodies (Table S5), cell lines (Table S6) and plasmids (Table S7) used in this study are given as supplemental material. The RRID portal (https://scicrunch.org/resources (accessed on 10 April 2020)) was used to provide antibody identifiers [30]. The anti-SFXN1 antibody HPA063745 was used for IP. For WB, either HPA063745 or HPA019543 anti-SFXN1 antibodies were mainly used in this study and gave similar results. Those two antibodies were validated for WB analysis on protein extracts from SFXN1-depleted human cells.

## 3. Results

### 3.1. SFXN1 Is an Evolutionarily Conserved Mitochondrial Protein Widely Distributed over Human Cell Lines

**Figure 1:**
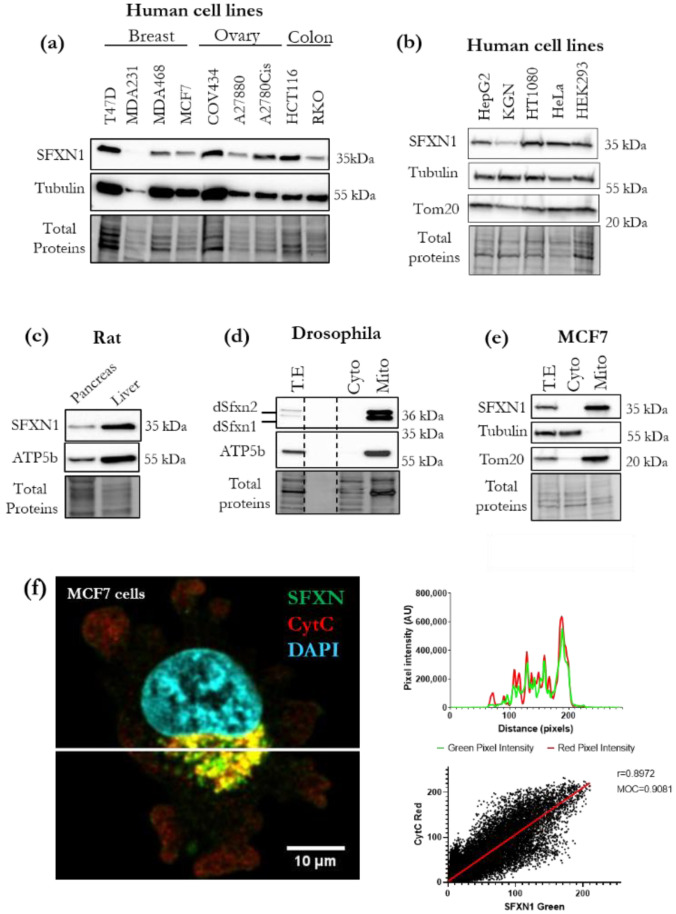
SFXN1 is a mitochondrially localized protein that is highly abundant in human cell lines. (**a**–**c**) Western blot analysis of SFXN1 levels in commonly used human cell lines or rat tissues. Total proteins were revealed using stain-free imaging of the membrane before immunodetection of SFXN1 with a commercial rabbit antibody (Sigma-Aldrich Cat# HPA019543, RRID:AB_1856789). Tubulin, Tom20 and the β subunit of the F1F0-ATPase (ATP5b)) served as a loading control. (**d**,**e**) Cell fractionation was performed on extracts from wild-type *Drosophila* larvae (**d**) and MCF7 cells (E). Total extracts (T.E.), cytosolic (Cyto) and mitochondrial (Mito) fractions were subjected to immunoblot analysis using the same anti-SFXN1 antibody as in panel a. ATP5b and Tom20 served as mitochondrial controls. (**f**) Sum projection of three consecutive stacks of MCF7 cell with SFXN1 (green) and cytochrome c (red) mitochondrial labeling on the left. One cell is shown; Figure S2c for the whole field. Signal distribution profile of green and red pixels, corresponding to the white horizontal bar on the left image, is shown on the right (upper panel), with highly similar behaviors. Correlations of green versus red intensities (black dots) corresponding to three individual projected stacks are also presented (on the right, lower panel). Pearson correlation coefficient (r) and best fitting line (red line) are shown; correlation is highly significant (*p* < 0.0001 two-tail test). Manders’s overlap coefficient (MOC) is also expressed. Both values, r and MOC, being high indicate a strong and significant colocalization between SFXN and Cyt *c* staining.

To identify a starting biological material suitable for the search of SFXN1 physical partners, we first compared the steady-state levels of the SFXN1 protein in different human cell lines. The specificity of this anti-SFXN1 antibody was confirmed using RNAi to deplete SFXN1 (Figure S1). SFXN1 was easily detected with Western blot in cell extracts from human transformed or tumor-derived cell lines (Figure 1a,b). We also tested our SFXN1 antibody in rat and *Drosophila*, two widely used organism models. Rat SFXN1, as well as dSfxn1/dSfxn2, the two *Drosophila* sideroflexins, were successfully detected (Figure 1c,d). There are several presentations of evidence for a mitochondrial localization of sideroflexins [4,9,15,17], and recently, a sub-localization of SFXN1 at the inner mitochondrial membrane (IMM) was confirmed [19]. Consistent with these findings, we found that SFXN1 is present in mitochondrial-enriched fractions from human MCF7, A2780 and COV434 cells and rat PC12 cells, as well as in mitochondrial fractions obtained from *Drosophila* larvae (Figure 1d,e and Figure S2). In addition, both endogenous SFXN1 and exogenous FLAG-tagged SFXN1 colocalized with mitochondria in our immunofluorescence studies (Figure 1f, Figures S2 and S3). All these results are in agreement with the data available in the Human Atlas Protein database [31], where a punctate pattern resembling that of mitochondria is seen using a specific antibody raised against the C-terminus of SFXN1 (Figure S4). This extremity was first predicted to be located in the intermembrane space of mitochondria [32], but recently, biochemical studies suggest instead that the C-terminus of SFXN1 is located inside the mitochondrial matrix [19].

### 3.2. Identification of SFXN1 Partners by CoIP-MS/MS

**Figure 2:**
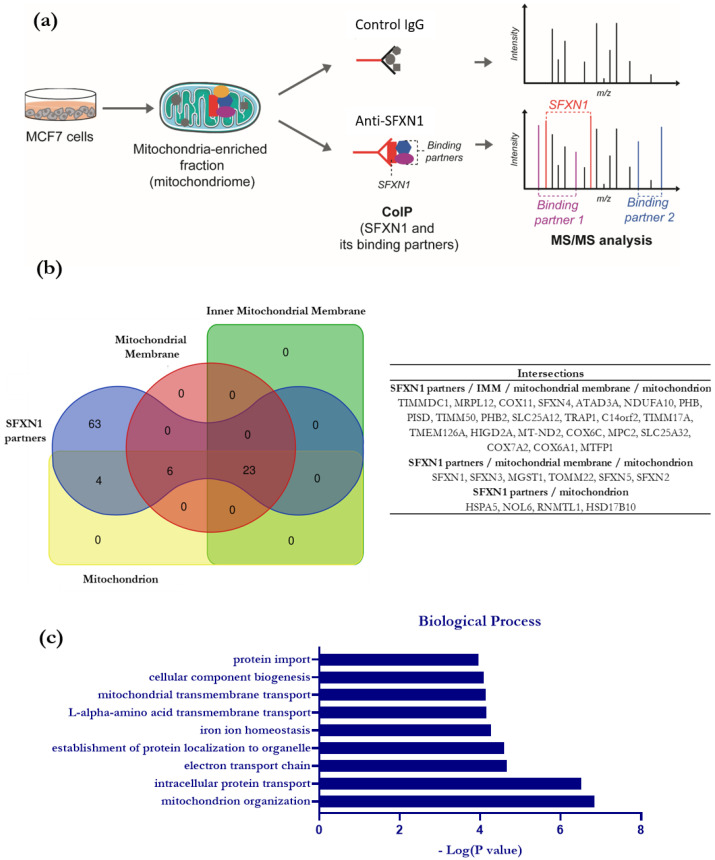
High-throughput search for SFXN1 physical partners (CoIP-MS) and related biological pathways. (**a**) Scheme of the methodology chosen to identify SFXN1 binding partners. An anti-SFXN1 antibody (Atlas Antibodies Cat# HPA063745, RRID:AB_2685111) was used to co-immunoprecipitate SFXN1 and its binding partners from MCF7-derived mitochondrial fractions. A control IP was performed using a non-specific rabbit IgG. Proteins identified by MS/MS solely in the SFXN1 immunoprecipitation and not in the control were listed as potential SFXN1 binding partners. (**b**) Venn diagram showing the overlaps between the potential SFXN1 partners and the mitochondrial proteins identified with MS/MS. Mitochondrial proteins were identified based on the Gene Ontology Annotation tool. (**c**) Biological processes related to the potential partners of SFXN1 determined using PANTHER Overrepresentation test.

To investigate the SFXN1 interactome, we launched a high-throughput search for SFXN1 physical partners using immunoprecipitation coupled with tandem mass spectrometry (MS/MS). The MCF7 human cell line was chosen because of its high SFXN1 content and ease of propagation, allowing for the recovery of a large number of cells as needed for fractionation and mitochondrial enrichment. First, SFXN1 was immunoprecipitated from mitochondria-enriched extracts of MCF7 cells. Proteins from the eluates were then identified with MS/MS. To ensure the specificity of the interactions, a control IP was performed with a non-specific IgG control (Figure 2a and Figure S5). The comparison of the proteins identified in the SFXN1-IP and IgG-IP allowed us to establish a list of 96 candidates. Analysis using STRING identified that 33 of them were mitochondrial components, among which 23 were annotated as proteins from the IMM (Figure 2b and Table S1).

Biological processes related to SFXN1 potential partners were then identified using Panther (Figure 2c, Tables S2 and S3). Among these, the electron transport chain (ETC) is significantly enriched, with seven proteins specifically found in the SFXN1-IP. As shown in Table S3, 3 subunits of complex I (TIMMDCI from the ND1 module, MT-ND2 and NDUFA10 from the ND2 module) and 3 subunits (COX6A1, COX6C and COX7A2) over the 13 forming complex IV and the complex IV assembly protein COX11 were co-immunoprecipitated with SFXN1. These results suggested a physical proximity between SFXN1 and the ETC. However, biochemical studies are required to confirm this hypothesis since only one peptide was found for these potential partners in the SFXN1-IP.

### 3.3. In Silico Analysis of the Interactome of SFXN1

#### 3.3.1. STRING Analysis

**Figure 3:**
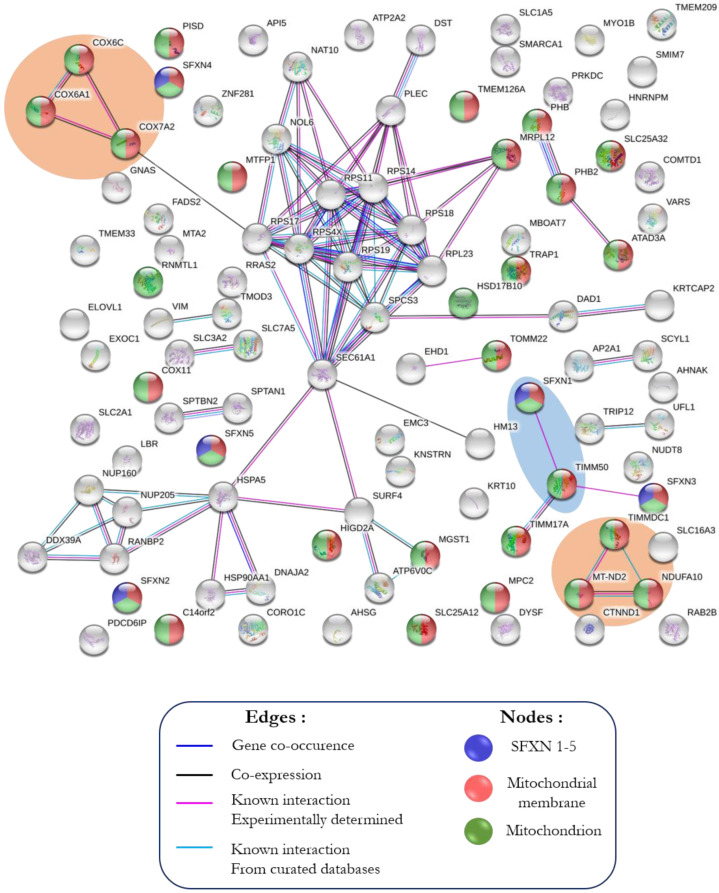
Analysis of the SFXN1 interactome using STRING. STRING protein–protein interaction network (https://string-db.org (accessed on 1 November 2019)) was constructed for physical partners found in our co-IP-MS/MS experiment. Two strong clusters are found between proteins of the electron transfer chain (complexes I and IV, in the orange areas) and one cluster involving TIM50 and SFXN1 is highlighted (in the blue area). SFXN family members, mitochondrial proteins and mitochondrial membrane proteins are colored in blue, green and red, respectively.

The physical partners listed in Table S1 were analyzed using the STRING v11.0 software [29]. The SFXN1 network shows three strong clusters, one of them containing SFXN1 (Figure 3). Functional enrichment analysis identified Gene Ontology (GO) terms related to oxidative phosphorylation, ribosome, RNA binding and transporter activity (Figures S6 and S7). Interestingly, this analysis confirmed the physical interaction between SFXN1 and TIM50 that was previously observed in large-scale studies [33,34].

#### 3.3.2. Metascape Analysis

- Enrichment Analysis and Clustering

**Figure 4:**
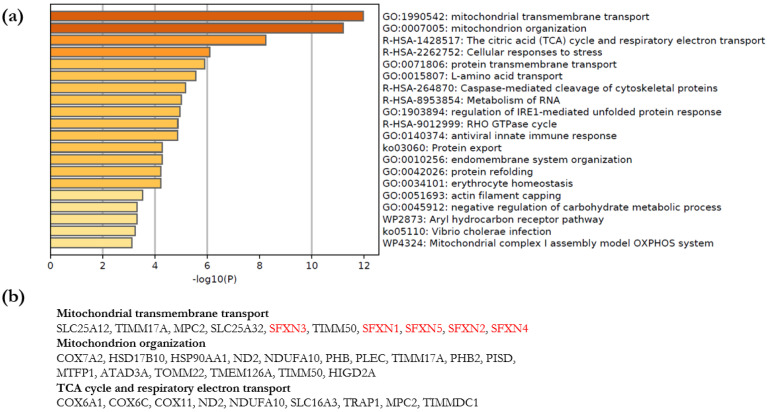
Metascape Enrichment analysis for SFXN1 partners. (**a**) Metascape bar graph for viewing top non-redundant enrichment clusters, one per cluster, using a discrete color scale to represent statistical significance (dark orange for the most significant clusters). (**b**) Hits from the top 3 enrichment clusters (mitochondrial transmembrane transport, mitochondrion organization, TCA cycle and respiratory chain).

The web-based portal tool Metascape was used to facilitate the interpretation of our data [35]. Metascape Express analysis consists of an automated analysis workflow beginning with identifier conversion and followed by gene annotation, membership search and enrichment analysis. The automated analysis of gene annotations using Metascape Express analysis permitted us to identify significantly enriched terms linked to our 96 candidates. The top 20 enriched clusters are shown in Figure 4. Interestingly, the top three enriched clusters are related to mitochondrial transmembrane transport, mitochondrion organization and mitochondrial respiration.

**Figure 5:**
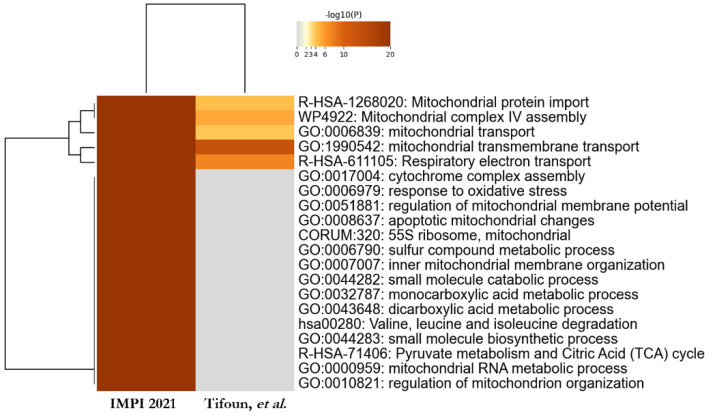
Comparison of top 20 enriched terms related to mitochondrial proteins and SFXN1 partners. Heatmap of enriched terms across input gene lists, colored by *p*-values (grey cells indicate the lack of enrichment for that term in the corresponding gene list) [3].

Because we immunoprecipitated SFXN1 from mitochondria enriched fractions, it is not really surprising to retrieve processes related to mitochondrial functions. Thus, we next tried to identify the processes related to mitochondrial functions that are preferentially enriched in our experimental dataset. For this purpose, a Metascape meta-analysis was performed to compare enriched terms associated with SFXN1 partners and those related to proteins known to be mitochondrial. As shown in Figure 5, among the most significant clusters related to mitochondrial proteins, five were significantly enriched for SFXN1 partners: mitochondrial transmembrane transport, respiratory electron transport, mitochondrial complex IV assembly, mitochondrial protein import and mitochondrial transport. Thus, potential SFXN1 physical partners identified in this study appear to be mainly involved in respiration and mitochondrial transport.

- Protein–protein interactome network analysis using Metascape

**Figure 6:**
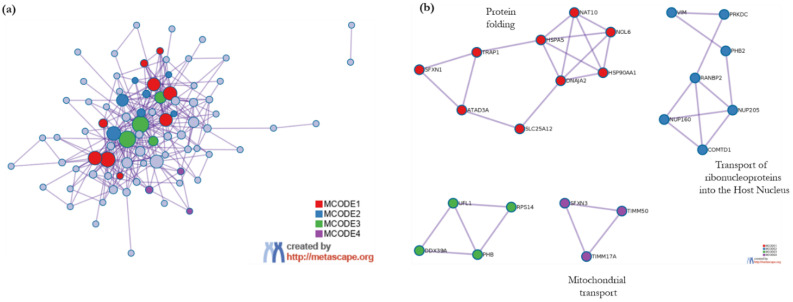
(**a**) Metascape visualization of the network formed by SFXN1 partners identified in our IP-MS/MS experiment. Densely connected complexes identified by the MCODE algorithm are colored according to their identities. (**b**) Four MCODE protein complexes extracted from the SFXN1 network. Functional annotations based on the top three functional enriched terms were available for MCODE1, MCODE 2 and MCODE4 complexes.

Metascape was utilized to automatically construct an SFXN1 protein–protein network from our list of putative SFXN1 physical partners based only on physical interactions available in the BioGrid, OmniPath, InWeb_IM and STRING databases. The protein interaction network formed by the putative SFXN1 physical partners is shown in Figure 6. Four densely connected protein complexes were identified, and among them, three were functionally labeled. SFXN1 and SFXN3 were found in two separate MCODE complexes respectively related to protein folding and mitochondrial transport. SFXN1 and ATAD3A were found in the same densely connected complex, highlighting the existence of a physical interaction between SFXN1 and ATAD3A, in agreement with our mass spectrometry analysis.

### 3.4. Comparative Study to Identify Highly Probable SFXN1 Partners

**Figure 7:**
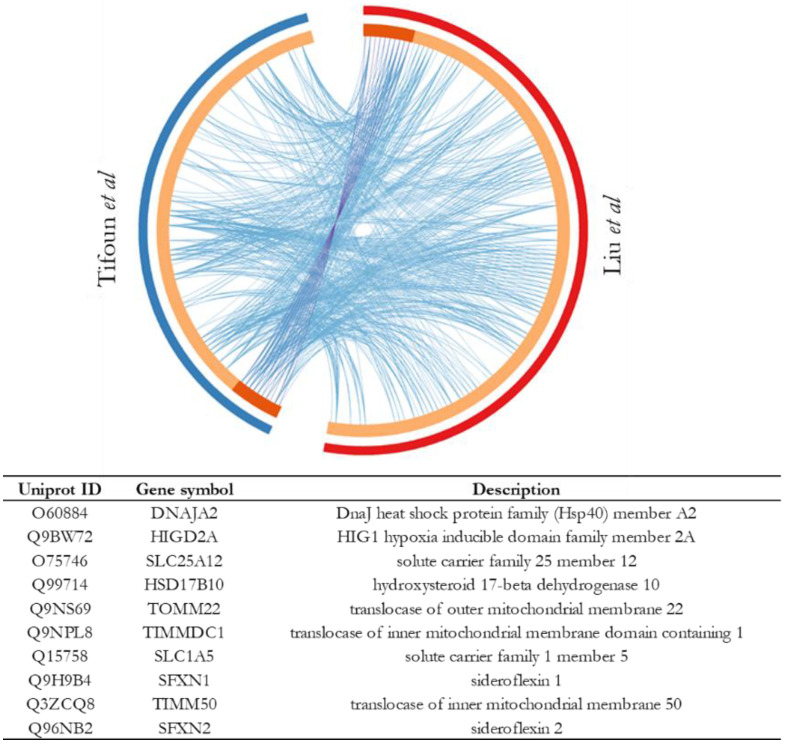
Circos plot showing overlapping genes from this study and Liu et al. [33]. On the outside, each arc represents a gene list. On the inside, dark orange represents the genes that are shared by the two lists, and light orange represents genes that are unique to a gene list. Purple lines link the same gene shared by the two lists. Blue lines link different genes that fall under the same ontology term. The table gives the list of shared genes.

In order to identify highly probable SFXN1 physical partners, we further utilized Metascape to compare our list of physical partners with the data from a combined affinity purification (AP) and BioID study in which SFXN1 was used as a bait and gave a list of 130 interactants [33]. Our comparative analysis highlighted ten proteins shared by the two lists including SFXN1, and among them were found SFXN2, 17β-HSD10, three mitochondrial translocases and the two metabolite carriers SLC25A12 and SLC1A5 (Figure 7). SLC25A12, also known as Aralar, is a calcium-binding mitochondrial carrier protein and is involved in the exchange of cytosolic aspartate for mitochondrial glutamate across the IMM [36]. SLC1A5 is a neutral amino acid transporter located in the plasma membrane. Thus, it was quite surprising to find this carrier as a shared protein. However, it was recently shown that a novel variant of the SLC1A5 gene transcribed from its alternative transcription initiation site was targeted to the mitochondria. This SCL1A5 variant is a mitochondrial glutamine transporter that is also able to mediate alanine and serine uptake in vitro [37,38].

Of note, whereas ATAD3A and TMEM126B were identified in our study, ATAD3B and TMEM126B were shown to interact with SFXN1 using the AP and BioID approach [33]. Perhaps these discrepancies could be explained by the shared peptides detected in mass spectrometry experiments.

Since SFXN1 was recently described as the mitochondrial transporter of serine linking SFXN1 to OCM, it is interesting to find serine hydroxy methyl transferase 2 (SHMT2) in Liu et al.’s list of putative SFXN1 partners. SHMT2 is the main mitochondrial enzyme of the OCM pathway, catabolizing serine into glycine following its entry inside the mitochondrion. However, we did not find this protein in our list of candidates.

**Figure 8:**
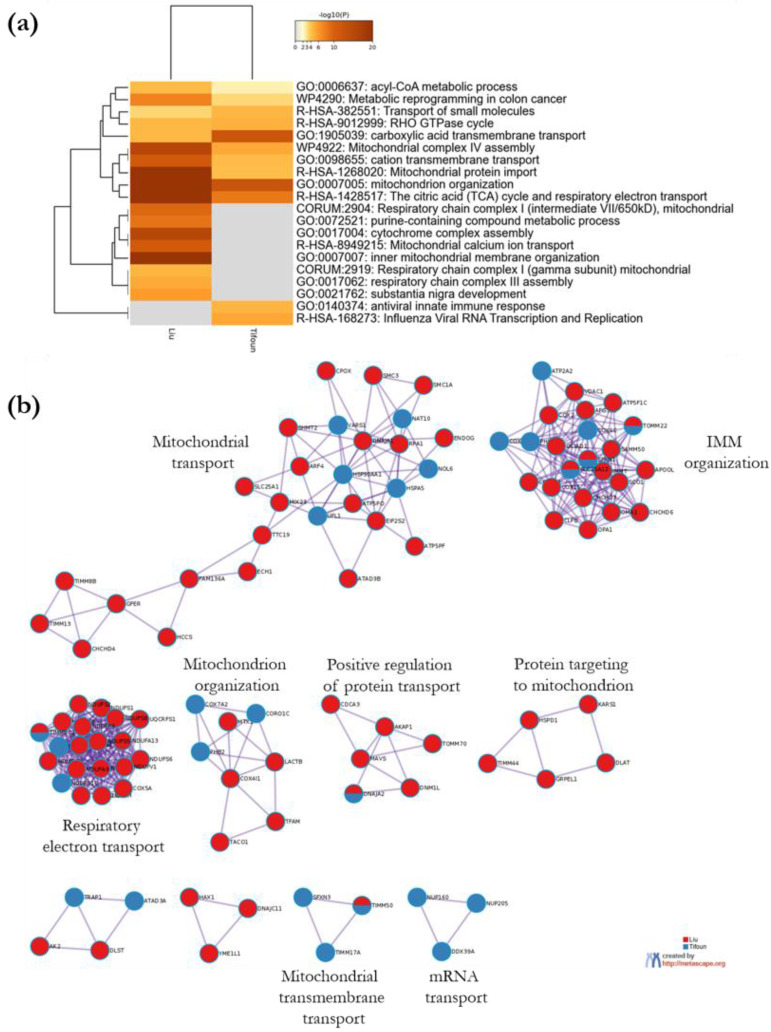
Visualization of the meta-analysis results based on the two lists of SFXN1 binding partners. (**a**) Heatmap showing the top enrichment clusters colored according to their significance. The term with the best *p*-value within each cluster is given as its representative term. (**b**) Metascape visualization illustrating densely-connected protein–protein interaction networks automatically identified from the merged lists of SFXN1 physical partners. A representative GO term was chosen between the top three best *p*-value terms to depict each MCODE network, when available. Network nodes are displayed as pies. Color code for pie sector represents a gene list (blue for our list, red for Liu et al.’s list).

To identify pathways that are enriched both in Liu et al.’s dataset and ours, we further analyzed the meta-analysis results obtained with Metascape. Ten enrichment clusters are shared between the two lists, the most significant ones being related to mitochondrial respiration, mitochondrion organization and mitochondrial transport (Figure 8a). A protein–protein interaction network was automatically constructed from a merge list including SFXN1 putative partners identified in both studies, and MCODE components were identified from the merged network (Figure 8b). Five shared genes were seen in the densely connected networks given by Metascape. For example, SFXN1 was found in a dense network described by the GO term “inner mitochondrial membrane organization”, together with SLC25A12 and TOMM22.

### 3.5. ATAD3, 17β-HSD10, TIM50 and NDUFA10 Physically Interact with SFXN1

**Figure 9:**
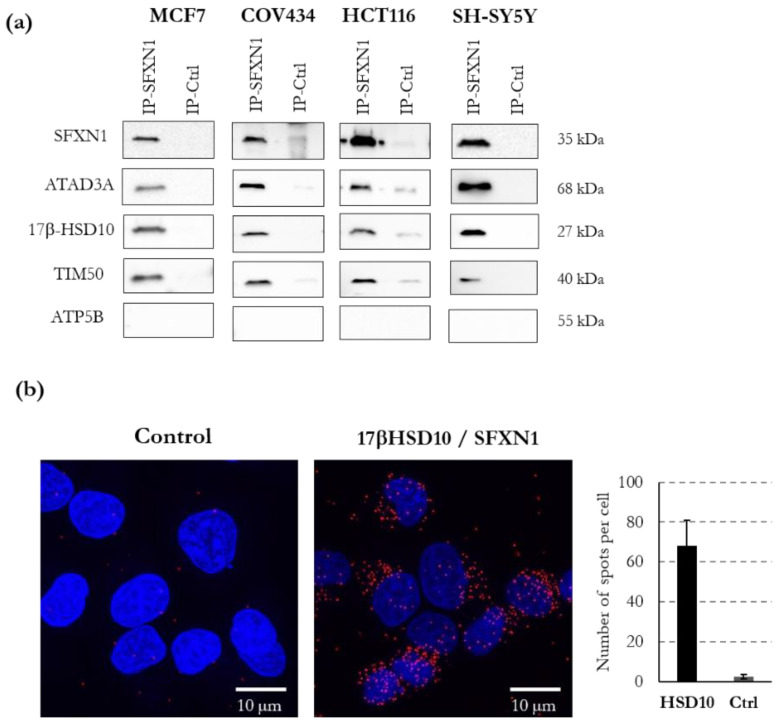
ATAD3, 17β-HSD10 and TIM50 are physical partners of SFXN1. (**a**) CoIP of ATAD3A, 17β-HSD10 and TIM50 using an anti-SFXN1 antibody on mitochondrial enriched fractions of MCF7, COV434, HCT116 and SH-SY5Y cells. An isotypic control (rabbit IgG) was used to ensure the specificity of the SFXN1 coIP. No coIP of the β subunit of the F1F0 ATPase (ATP5B) was seen and thus serves as a negative control. (**b**) Proximity ligation assay showing the physical vicinity of SFXN1 and 17β-HSD10 in MCF7 cells (red dots). Signal quantification regarding the control condition (without primary antibodies) is reported on the right panel. Two independent experiments gave similar results. Scale bar: 10 μm.

Next, we investigated whether the interactions between SFXN1 and the candidates of particular interest could be confirmed. Foremost, we considered those with a high Mascot’s score. Since the steady-state levels of SFXN1 may be decreased in neurodegenerative diseases and its closest homologue SFXN3 may regulate synaptic morphology [7,9], we focused on candidate proteins that had been linked to neurological or neurodegenerative disorders. For example, pathological mutations in ATAD3A and 17-beta-HSD10 have been previously reported [39,40]. Thus, we analyzed by immunoblot whether ATAD3A and 17β-HSD10 were present in SFXN1 immunoprecipitates (SFXN1-IP). ATAD3A and 17β-HSD10 were specifically detected in the SFXN1-IP in all the cell lines tested, while ATP5b was not detected, confirming the relevance of the MS/MS analysis (Figure 9a and Figure S5). Additionally, a proximity ligation assay confirmed the close vicinity of SFXN1 and 17β-HSD10 (Figure 9b). TIM50 and NDUFA10 (not shown) were also confirmed as SFXN1 physical partners.

## 4. Discussion

SFXN are mitochondrial carriers that are emerging as the main actors in OCM and iron metabolism. Nevertheless, the precise functions of these proteins remain unclear. Whereas SFXN1 has recently been identified as the mitochondrial transporter of serine, it may also import other neutral amino acids such as Ala, Cys and Gly) or metabolites inside the mitochondria [17]. In this study, we investigated the SFXN1 interactome via the mass spectrometry analysis of coimmunoprecipitated proteins, followed by in silico analyses of the candidates. To our knowledge, this study is the first study focusing on the identification of SFXN1 physical partners. In agreement with the previously suggested role of SFXN in the regulation of mitochondrial respiration [6,16,19], we identified some subunits of the mitochondrial ETC. Moreover, a physical interaction between SFXN1 and ATAD3A, 17β-HSD10 or TIM50 was further confirmed using different human cell lines.

### 4.1. Biological Processes Linked to SFXN1 Interactome

Here, we identified mitochondrial organization, mitochondrial transport and mitochondrial respiration as the main biological processes linked to the SFXN1 interactome. This does not seem very surprising since SFXN1 is inserted in the IMM and probably in the close vicinity of key functional complexes such as respiratory complexes. Thus, we do not exclude the hypothesis of a co-immunoprecipitation of members of respiratory complexes because of their physical proximity with SFXN1. Whether SFXN1 directly interacts with some subunits of the respiratory complexes needs to be further investigated. Nevertheless, several studies on KO cells reported a regulatory role of SFXN in mitochondrial respiration (for review see [3]). In agreement with these findings, we observed that the knockdown of SFXN1 in MCF7 cells impaired the basal and maximal OCR, implying defective mitochondrial respiration (Figure S8). Thus, SFXN1 levels must be tightly controlled to maintain mitochondrial respiration. Previously, we reported a slight increase in iron mitochondrial levels that may be the consequence of a defective biosynthesis of heme or iron–sulfur cluster, two types of cofactors found in some subunits of the respiratory chain. Thus, we wondered if some regulators of iron homeostasis would co-immunoprecipitate with SFXN1. Over the 83 proteins related to iron ion homeostasis in PANTHER, only SFXN1-5, which were annotated as putative transporters of a metabolite involved in iron homeostasis, were found in SFXN1-IP (Table S3). Since late 2018, SFXN1 has been known as the mitochondrial transporter of serine, and SFXN2, SFXN3 and SFXN5 may also ensure this function [17].

### 4.2. SFXN1 May Interact with the Other Members of the SFXN Family

Whether SFXN1 interacts with others SFXN to form multimers remains an open question. This possibility cannot be excluded since several examples of amino acid transporters functioning as heterodimer exist, such as the x_c_^-^ system (a Cys-Glu antiport). Nevertheless, it is also possible that our SFXN1 antibody was able to interact with SFXN2, SFXN3 and SFXN5 and to a lesser extent with SFXN4 under native conditions. Indeed, the order of the SFXN1 homologues in the list of MS/MS proteins is in accordance with their degree of similarity, with SFXN1 and SFXN3 being the most closely related while SFXN4 is the most divergent. Nevertheless, the question of the multimerization of SFXN merits attention as SFXN1 has recently been shown to form a complex of 130 kDa in a BN-PAGE analysis of mitochondria isolated from HEK human cells [41].

### 4.3. SFXN1, ATAD3A and 17β-HSD10

Here, we identified ATAD3A and 17β-HSD10 as physical interactors of SFXN1. ATPase family AAA domain-containing protein 3 A (ATAD3A) is a mitochondrial transmembrane protein playing a key role in the maintenance of the mitochondria–endoplasmic reticulum (ER) contacts as well as the regulation of mitochondrial biogenesis and dynamics [42]. ATAD3A associates with different components of the IMM, including OXPHOS complex I and the prohibitin complexes [43]. Our results suggested that ATAD3A and SFXN1 belong to the same multimeric complexes. Mutations in the *ATAD3* human gene cluster were reported to cause neurological diseases (Harel–Yoon syndrome, OMIM entry # 617183) and more specifically cerebellar disorders [39,44]. Its loss in mouse conditional knockout models leads to severe brain defects [43]. Interestingly, ATAD3 depletion in worms induces mitochondrial iron accumulation and alters the expression of iron-regulating genes [45]. Mitochondrial iron overload has also been reported as a consequence of *SFXN2* or *SFXN4* gene knockout [15,16], and we previously observed a slight increase in mitochondrial iron levels when SFXN1 levels were decreased by RNAi [3]. Whether ATAD3 and SFXN1 participate in the same pathways deserves further investigation.

17β-HSD10 (also known as 3-hydroxyacyl-CoA dehydrogenase type-2, ERAB, ABAD or MRPP2) is a multifunctional NAD(+)-dependent dehydrogenase involved in the biosynthesis of steroids and neurosteroids in the mitochondrial matrix [46]. It is also one of the three subunits of the mt-RNAse P complex essential for the maturation of mitochondrial RNAs. Pathological mutations in 17β-HSD10 were described in a X-linked disorder [40] (HSD10 mitochondrial disease, OMIM entry #300438). 17β-HSD10 may also be involved in AD through its interaction with the β-amyloid peptide or its role in neuroactive steroid metabolism [47,48]. In agreement with our findings, a physical interaction between SFXN1 and 17β-HSD10 was previously reported by Liu et al., reinforcing the hypothesis of a close proximity between SFXN1 and 17β-HSD10. It will thus be interesting to further investigate the relationships between 17β-HSD10 and SFXN1. More specifically, whether SFXN1 could regulate 17β-HSD10 functions i.e., steroid biosynthesis and mt-RNA maturation, remains an open question.

### 4.4. Physical Interaction between SFXN1 and Mitochondrial Translocases

SFXN proteins are synthesized on cytosolic proteins such as the 1123 nuclear-encoded mitochondrial proteins reported in MitoCarta 3.0 [49]. Mitochondrial proteins are imported through one of the five protein import pathways depending on their sequence, structure and final location inside the mitochondria [30]. The translocase of the outer membrane (TOM), the main entry door for the mitochondrially targeted proteins, consists of the Tom40 channel associated with several subunits including the receptors Tom20, Tom22 and Tom70. Once translocated through the TOM complex, IMM proteins are then directed to their final destination either by the TIM22 or TIM23 complex. The TIM22 complex preferentially delivers multi-spanning transmembrane proteins with internal targeting signals to the IMM, such as the metabolite transporters of the SLC25A family, whilst the TIM23 complex is rather dedicated to the translocation of IMM proteins containing a cleavable presequence [50,51]. Components of the human TIM22 complex include Tim22 itself, which forms the channel, several IMS chaperones shuttling between the TOM and TIM22 complex (namely, hTim9, hTim10, hTim10b), Tim29 [51] and AGK (acylglycerol kinase) [52,53]. In humans, the TIM23 complex consists of TIM17A/B and TIM23 subunits forming the channel, the TIM50 receptor and several other proteins. Recent studies highlight the role of AGK in the mitochondrial import of SFXN1, in agreement with its IMM insertion via the TIM22 complex, as expected for a mitochondrial metabolite carrier [19,41]. In our coIP-MS/MS experiment, we as expected identified TOM22, the central receptor of the TOM complex [54]. On the contrary, neither TOM20 (the receptor for presequence-containing proteins) nor TOM70 (the preferential receptor for IMS carriers) was detected, probably because of a too-transient interaction. However, the detection of the TIM23 complex components, TIM50 and TIM17a, is surprising since metabolite carriers like SFXN1 are rather supposed to reach the IMM through the TIM22. Because TOM22 and TIM50 can physically interact [55], we favor the hypothesis that we have immunoprecipitated a SFXN1-TOM22 complex containing TIM50 and TIM17A as indirect physical partners of SFXN1. Nevertheless, no component of the TIM22 complex specifically co-precipitated with SFXN1 in this study. One explanation could be that the epitope recognized by our immunoprecipitating antibody is masked when SFXN1 interacts with subunits of the TIM22 complex upon its translocation in the IMM.

### 4.5. Other Partners

Here, we focused on ATAD3A and17β-HSD10, but other candidates with a high Mascot’s score also merit our attention. Indeed, the interaction between SFXN1 and the microsomal glutathione S-transferase MGST1, a detoxification enzyme belonging to the membrane associated proteins in eicosanoid and glutathione metabolism (MAPEG) superfamily [56], could be further investigated. MGST1, an essential enzyme for development and hematopoietic differentiation [57], possesses a lipid peroxidase activity, such as the glutathione peroxidase and main regulator of ferroptosis GPX4.. Excess iron, as can be seen upon the downregulation of SFXN, may trigger oxidative stress, eventually leading to a ferroptotic cell death if lipid peroxides are not detoxified by enzymes with glutathione peroxidase activity [58]. SFXN1, as the mitochondrial transporter of serine may also indirectly participate in the biosynthesis of glutathione (tripeptide γ−L-Glu-L-Cys-Gly), allowing the import of serine in mitochondria and its catabolism by the SHMT2 enzyme, thereby releasing glycine. The functional significance of the SFXN1–MGST1 interaction observed in this study remains to be documented.

Previously, connexin32 and SCIRR69 were identified as physical partners of SFXN1 [59,60]. However, these proteins were not found on our list of partners. This could be related to methodological differences (basal or stress conditions for example) or cell-type-specific interactions. Finally, SFXN1 was recently identified as a SERAC1-interacting protein from a co-IP/MS analysis. SERAC1, a mitochondrial protein associated with MEGD(H)EL syndrome, may facilitate the transport of cytosolic serine into the mitochondria by interacting with and stabilizing SFXN1 [61]. SERAC1 is another previously described partner of SFXN1 which was not identified in our co-IP/MS analysis of SFXN1-interacting proteins. As stated in Fang et al. [61], SFXN1-SERAC1 interaction is also supported by a highthroughput BioID-based protein–protein interaction map (https://humancellmap.org/ (accessed on 13 August 2022)) [34]. In both studies, the SFXN1–SERAC1 interaction was identified in HEK293 cells overexpressing either SERAC1 [61] or SFXN1 [34]. In this study, we sought to uncover physiologically relevant interactions by immunoprecipitating endogenous SFXN1 from MCF7-derived mitochondrial extracts rather than SFXN1-overexpressing cells. Thus, the absence of SERAC1 in our SFXN1 interactome may be explained by either the different cell lines used (mammary tumor cells versus kidney embryonic cells) or a labile interaction that could not be detected with endogenous SFXN1. Thus, we do not exclude the possibility of a SERAC1–SFXN1 interaction and this point should be further investigated in our models.

## 5. Conclusions

The SFXN1 physical interactors identified here suggested that SFXN1 could be in the vicinity of respiratory complexes I and IV. SFXN4 was recently shown to act as an assembly factor for complex I, interacting with the mitochondrial complex I intermediate assembly (MCIA), which contains TMEM126B [62]. Amongst the FXN1 partners identified in this study, TMEM126A and TIMMDC1 are two complex I assembly factors interacting with MCIA [63,64]. Thus, it could be interesting to further investigate the role of SFXN1 in regulating complex I assembly. Whether SFXN proteins are able to form multimers is also an open question since we and others have found SFXN1 homologues in the SFXN1 interactome. Finally, in this study, evidence for the interaction of SFXN1 with ATAD3A, and with 17β-HSD10 was obtained via shotgun mass spectrometry and biochemical validations. However, further work is needed to understand the significance of these physical interactions and to shed light on the role of SFXN1 in neuronal pathophysiology.

## Data Availability

Proteomics data are available via ProteomeXchange with identifier PXD030749. The data that support the findings of this study are available from the corresponding author, N.L.F., upon reasonable request.

## Supplementary Materials

The following supporting information can be downloaded at: https://www.mdpi.com/article/10.3390/biology11091298/s1, Figure S1. Specificity of the anti-SFXN1 antibody used for immunoblotting; Figure S2. SFXN1 is a mitochondrial protein; Figure S3. FLAG-tagged SFXN1, SFXN2 and SFXN5 proteins colocalize with mitochondria; Figure S4. Immunostaining of MCF7 cells with the HPA063745 antibody reveals a mitochondrial pattern; Figure S5. SFXN1 is successfully immunoprecipitated from mitochondria-enriched fractions from MCF7 cells using a specific antibody recognizing its C-terminus (Atlas Antibody HPA063745, Sigma-Aldrich); Figure S6. Analysis of STRING v11.0-generated SFXN1 interactome reveals pathways linked to ribosome and OXPHOS; Figure S7. Analysis of STRING v11.0-generated SFXN1 interactome reveals molecular functions linked to transporter activity, COX activity and RNA binding; Figure S8. Knockdown of SFXN1 in MCF7 cells impairs mitochondrial respiration; Table S1. Proteins identified with MS/MS solely in the SFXN1 IP; Table S2. Biological processes related to the SFXN1 using PANTHER; Table S3. Proteins identified in each biological process; Table S4. Biological pathways related to the SFXN1 binding partners using REACTOME; Table S5. List of the antibodies used in this study; Table S6. Cell lines used in this study; Table S7. Plasmids used in this study (related to Figure S3, purchased from Genscript).
