# Supplementary material for: A High-Throughput Search for SFXN1 Physical Partners Led to the Identification of ATAD3, HSD10 and TIM50
Source: Biology (Basel). 2022 Aug 31;11(9):1298. doi: 10.3390/biology11091298 (PMC9495560; doi:10.3390/biology11091298)
Supplement: Supplementary file 1 [file biology-11-01298-s001.zip › biology-1865973-supplementary.pdf]

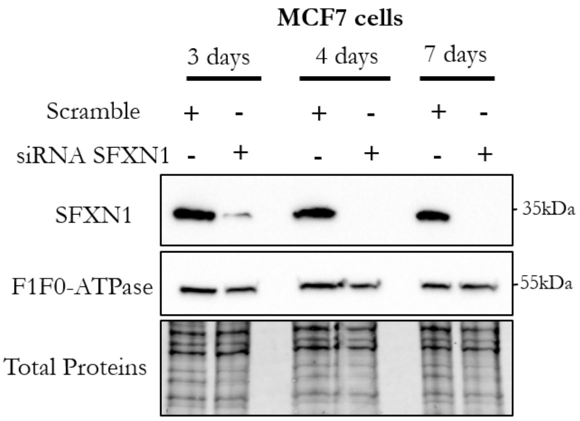

Figure S1. Specificity of the anti-SFXN1 antibody used for immunoblotting. MCF7 cells were transfected with a pool of SFXN1 siRNA (sc-91814, Santa Cruz Biotechnology), and protein extracts were prepared at 3, 4 and 7 days post-transfection. SFXN1 levels were assessed with Western blot using a rabbit anti-SFXN1 antibody (Sigma-Aldrich Cat# HPA019543, RRID:AB_1856789). The ATP5b subunit of the F1F0-ATPase is used as a loading control. Stain-free detection of total proteins is also shown.

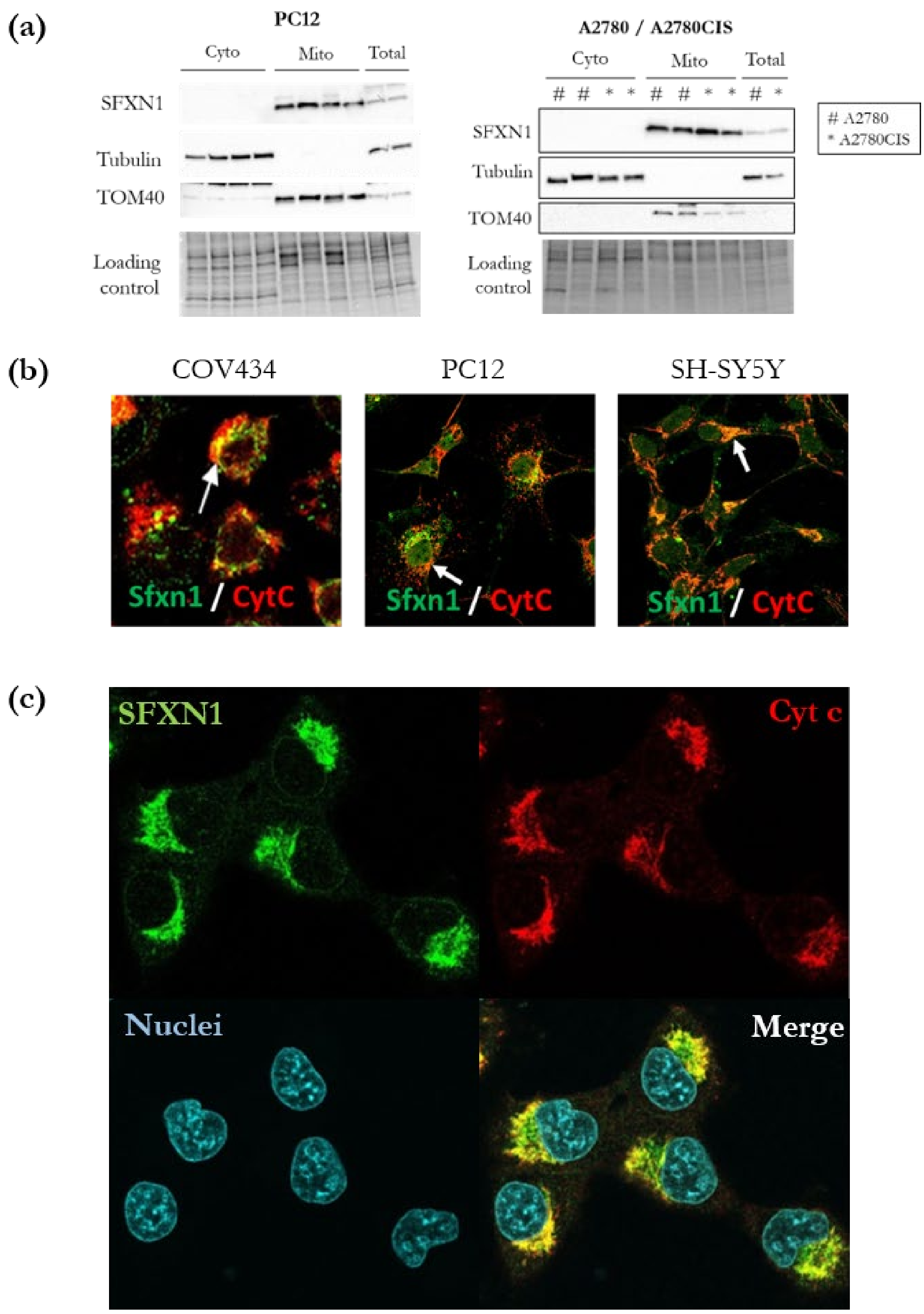

Figure S2. SFXN1 is a mitochondrial protein. (**a**). SFXN1 is detected in mitochondria-enriched fractions from PC12, A2780 andA2780CIS cells but not in cytosolic fractions. Western blot analysis of SFXN1 levels was performed with a rabbit specific anti-SFXN1 antibody (Sigma-Aldrich Cat# HPA019543, RRID:AB_1856789). TOM40 is shown to check the purity of the cytosolic fractions. As expected, Tubulin is only detected in cytosolic fractions, proving the absence of contamination of the mitochondrial fraction by cytosolic proteins. (**b**). SFXN1 colocalizes with cytochrome c in COV434, PC12 and SH-SY5Y. Immunostaining was performed with a rabbit specific anti-SFXN1 antibody (Sigma-Aldrich Cat# HPA019543, RRID:AB_1856789), and images were acquired by confocal fluorescence imaging. (**c**) Immunodetection of SFXN1 and cytochrome c (mitochondrial staining) in MCF7 cells. A representative image obtained by confocal imaging is shown.

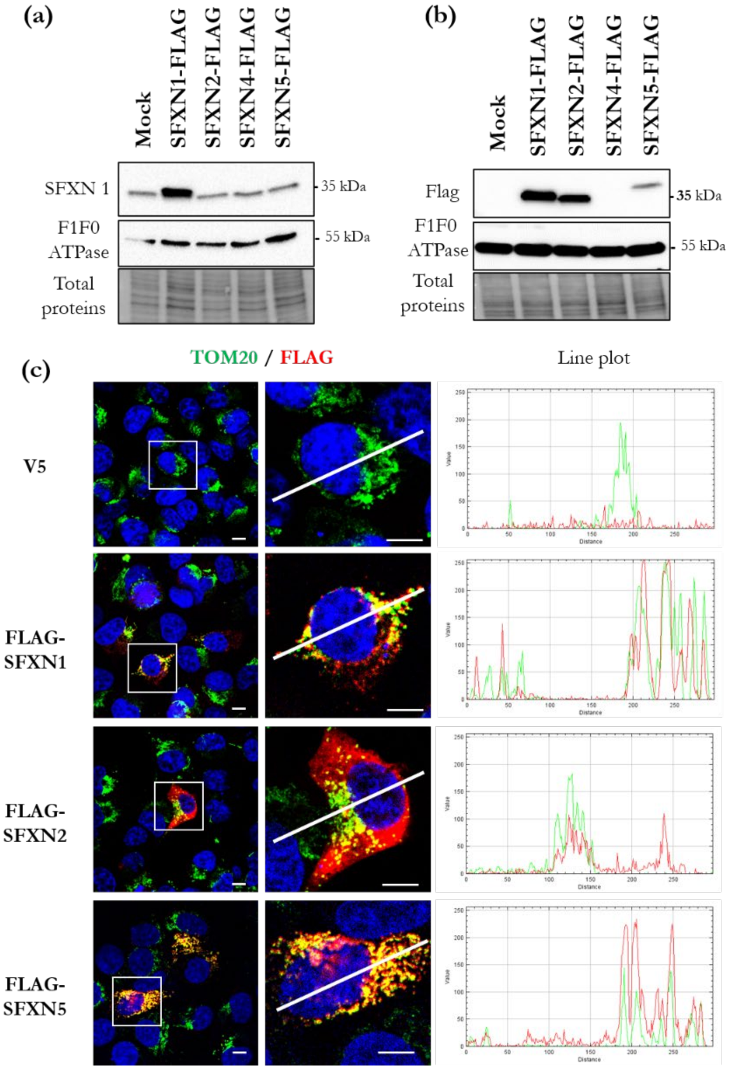

Figure S3. FLAG-tagged SFXN1, SFXN2 and SFXN5 proteins colocalize with mitochondria**.** (**a**,**b**) Transient transfection of HEK293 cells with SFXN expression vectors (Genscript). HEK293 cells were transiently transfected with an empty vector or plasmids encoding FLAG-tagged SFXN1, SFXN2, SFXN4 or SFXN5. Western blot analysis was performed using a polyclonal anti-SFXN1 antibody or an anti-FLAG antibody. Total protein amount was observed using stain-free labeling before SFXN1, FLAG and F1F0-ATPase revelation. (**c**) Immunofluorescence assay on MCF7 cells transfected with V5 tag or with different FLAG-SFXNs. Cells were stained for TOM20 (green) and exogenous SFXNs using anti-FLAG antibody (red). The fluorescence intensities along the lines are shown as line profile graphs. Scale bar: 10 μm.

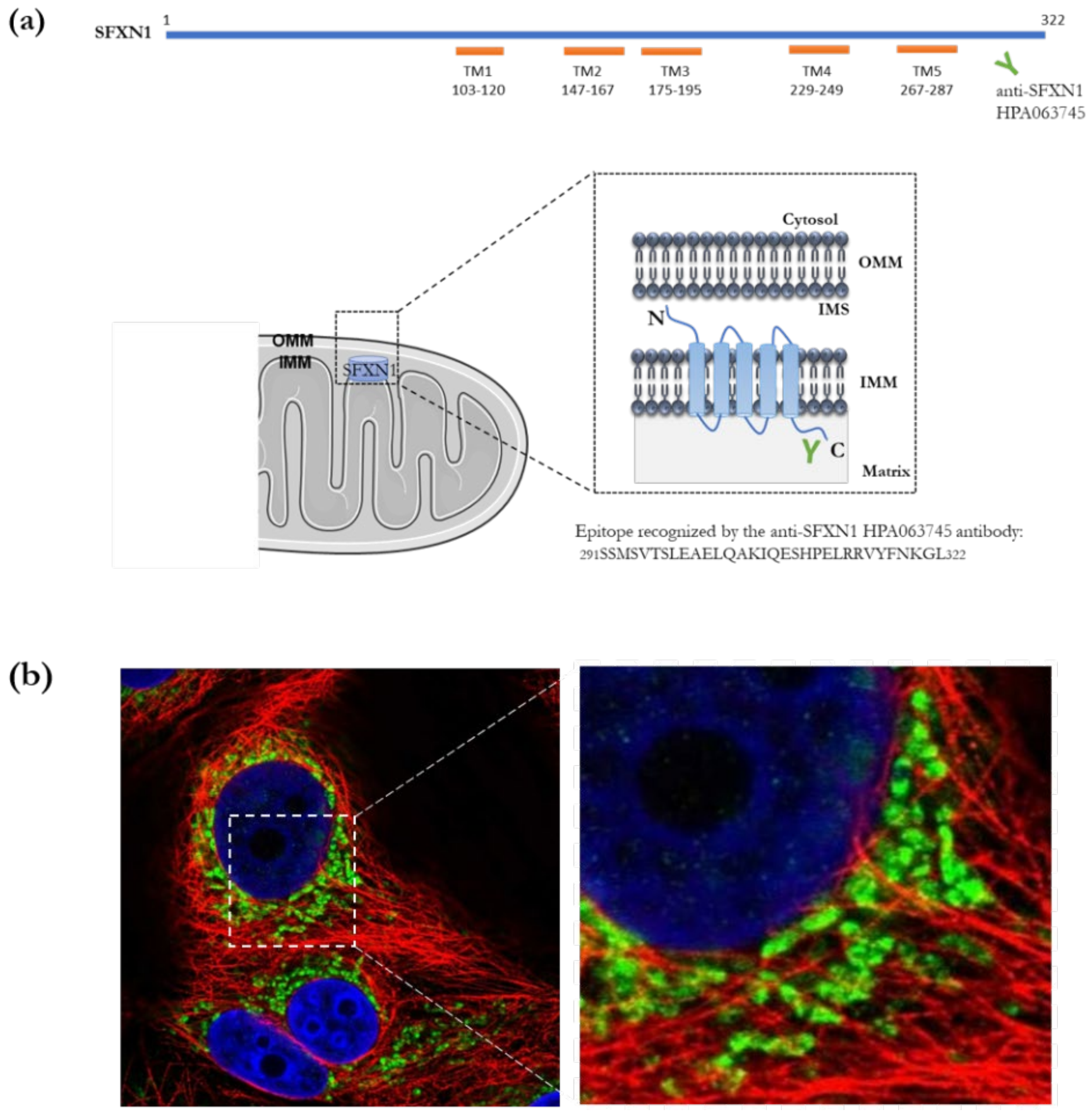

Figure S4. Immunostaining of MCF7 cells with the HPA063745 antibody reveals a mitochondrial pattern. (**a**) Scheme of human SFXN1 protein (1–322 amino acids) and its topology. The orange bars correspond to transmembrane helices (TM). The epitope recognized by the Atlas Antibody HPA063745 (Sigma-Aldrich) is present at the C-terminus of the SFXN1 protein (aa291-322, SSMSVTSLEAELQAKIQESHPELRRVYFNKGL), which is probably located in the mitochondrial matrix. (**b**) Immunostaining of MCF7 cells with the HPA063745 antibody reveals spots presumed to be mitochondria. Image credit: Human Protein Atlas, available at http://www.proteinatlas.org (accessed on 15 April 2020), image 63745/SFXN1/IF available from version v19.2.proteinatlas.org. Cells were stained using an anti-SFXN1 antibody (green) or an anti-microtubules antibody (red). Nuclei are stained in blue (DAPI). Scale bar: 10 µm.

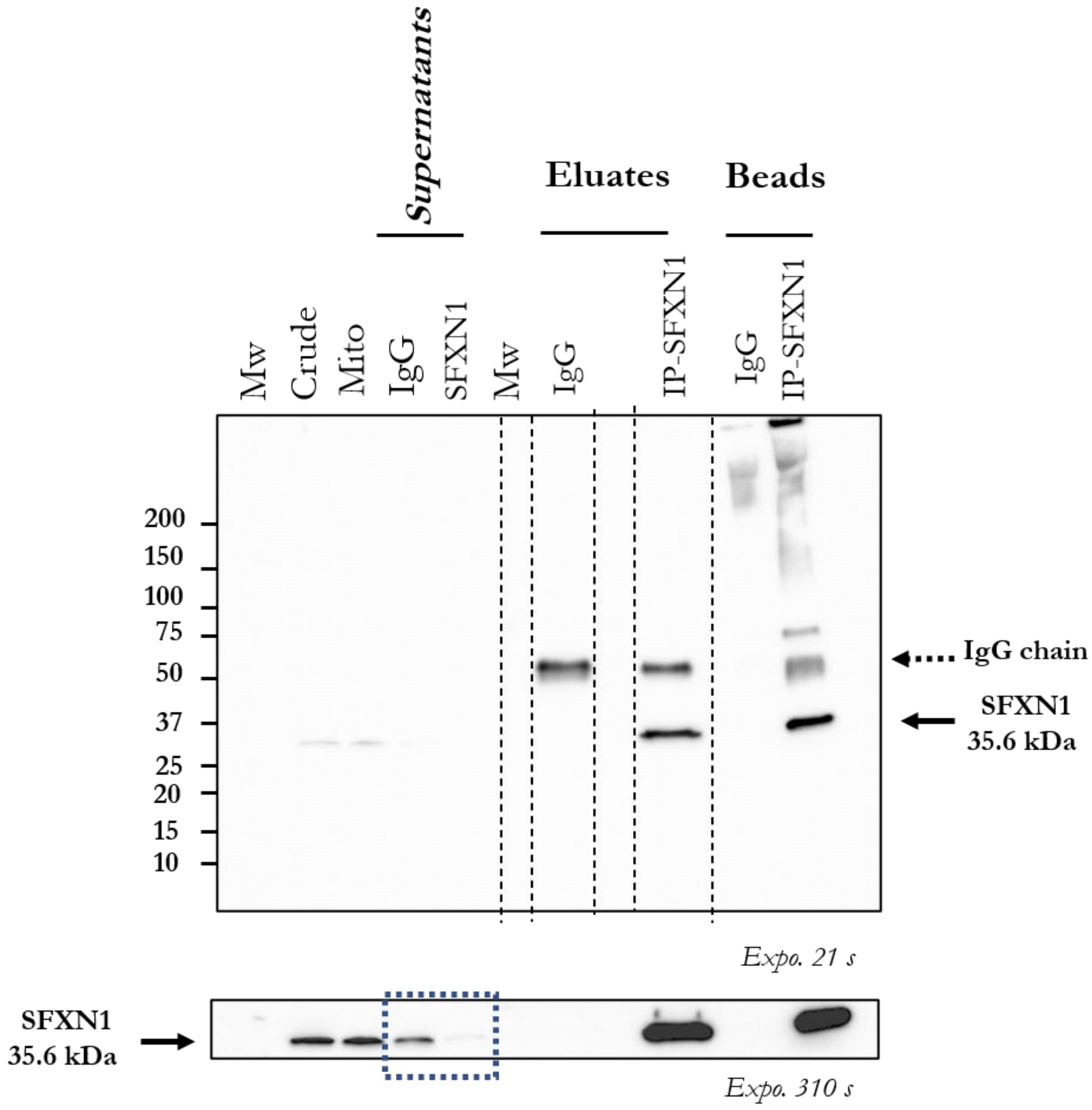

Figure S5. SFXN1 is successfully immunoprecipitated from mitochondria-enriched fractions from MCF7 cells using a specific antibody recognizing its C-terminus (Atlas Antibody HPA063745, Sigma-Aldrich). An isotypic control (anti-Rabbit IgG 12–370, Merck Millipore) was used to show the specificity of SFXN1 IP. After two rounds of elution, beads were conserved and loaded to control the efficiency of elution: a significant amount of protein remained on the beads. A longer exposure is provided to show the depletion of SFXN1 in supernatants from SFXN1 IP compared with control IgG IP.

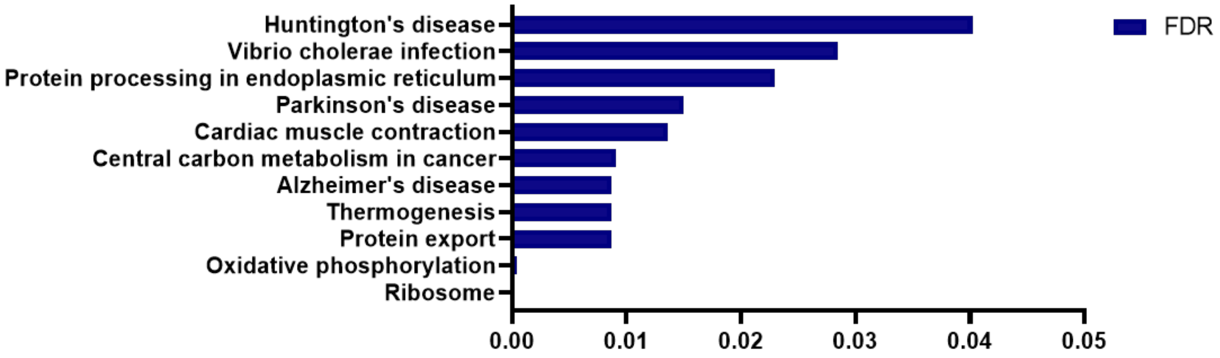

Figure S6. Analysis of STRING v11.0-generated SFXN1 interactome reveals pathways linked to ribosome and OXPHOS. KEGG pathways with a false discovery rate (FDR) below 0.05 are listed.

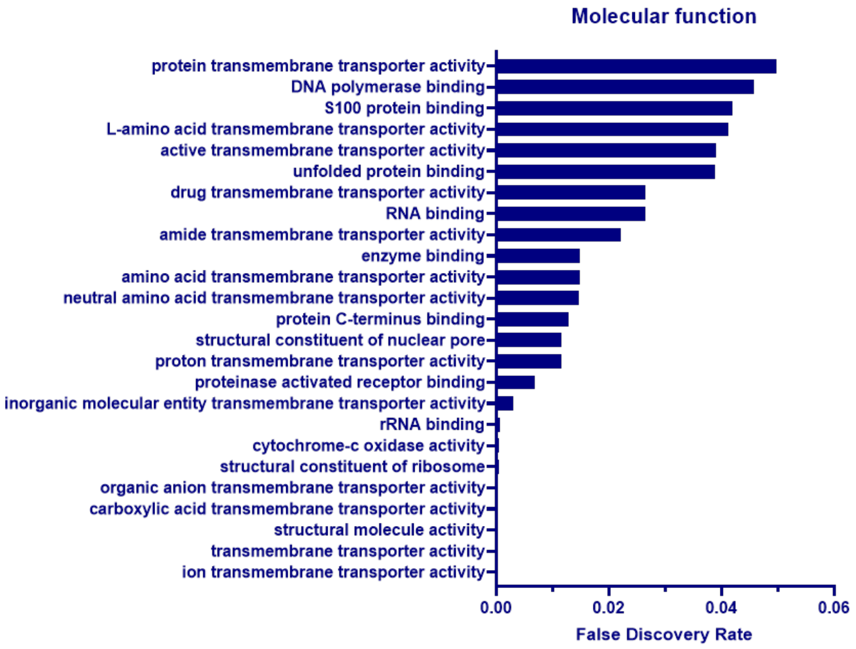

Figure S7. Analysis of STRING v11.0-generated SFXN1 interactome reveals molecular functions linked to transporter activity, COX activity and RNA binding. Molecular functions linked to SFXN1 physical partners are listed according to the FDR.

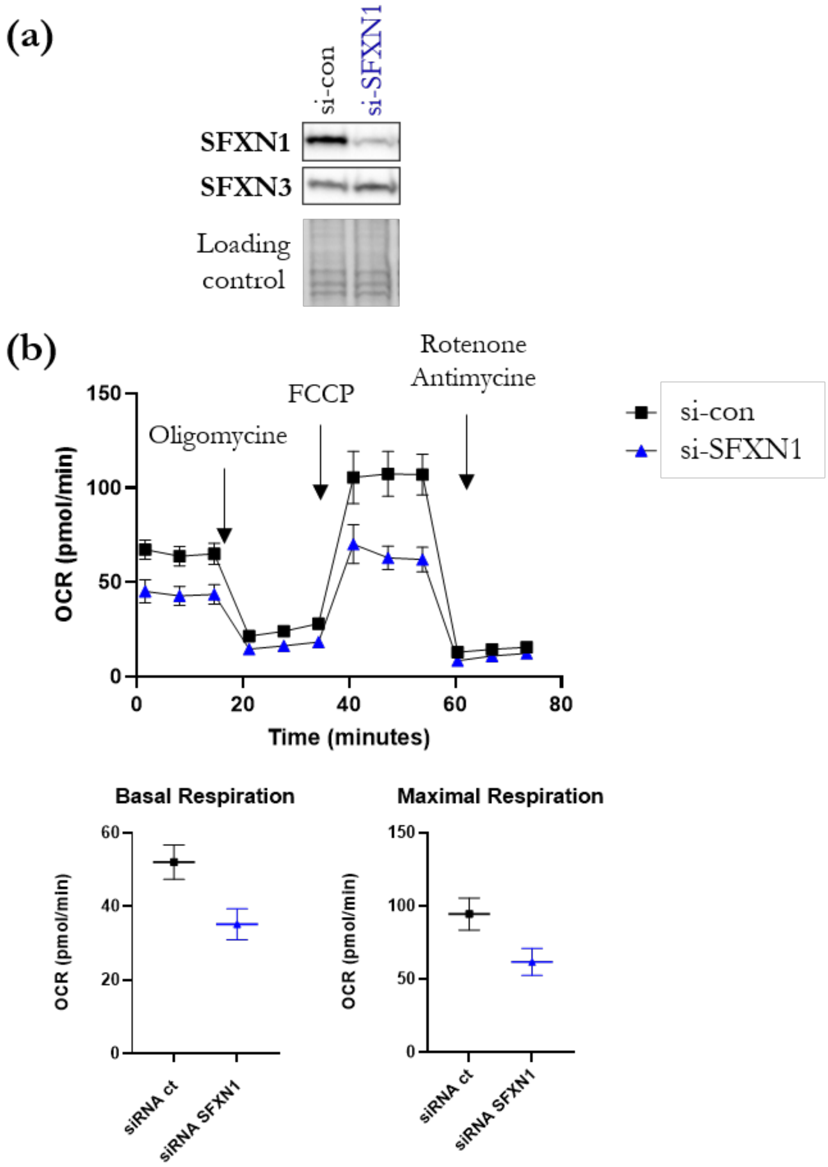

Figure S8. Knockdown of SFXN1 in MCF7 cells impairs mitochondrial respiration. (**a**) Validation of SFXN1 knockdown by Western blot. SFXN3 was also detected as a control of the SFXN1 siRNA specificity. si-con: siRNA with scrambled sequence; si-SFXN1: pool of three siRNA targeting SFXN1 transcripts. Stain-free detection of proteins served as a loading control. (**b**) Seahorse experiment to monitor oxygen consumption rate of transiently transfected MCF7 cells (Mito Stress Test, Agilent).

Table S1. Proteins identified with MS/MS solely in the SFXN1 IP.

Name	#Pept [1]	#PSM [2]	Score	Name	#Pept [1]	#PSM [2]	Score
**Sideroflexin-1**	11	17	605	CD98	1	1	39
**Sideroflexin-3**	10	11	367	Delta(6) desaturase	2	2	38
**Sideroflexin-2**	5	7	328	XLalphas	2	2	38
Vimentin	9	10	224	hnRNP M	1	1	38
MGST1	2	3	169	Desmoyokin	1	1	37
ATAD3A	5	5	124	COX6A1	1	1	36
**Sideroflexin-5**	2	2	123	RAB2B	2	2	36
GLUT-1	1	2	113	HSP 75	1	1	36
KCP-2	2	2	109	TMEM33	1	1	35
MRP-L12	1	1	96	Nucleoporin Nup205	1	1	35
17-beta-HSD 10	3	4	95	LPLAT 7	1	1	34
ValRS	2	2	94	PISD	1	1	34
hLAT1	1	1	89	SMARCA1	1	1	33
MPC2	1	1	89	Prohibitin-2	1	1	33
PIP5K1A-PSMD4	1	1	87	MFTC	1	1	33
Tropomodulin-3	1	2	84	COX11	1	1	33
RCF1b	1	1	81	Zinc finger protein 281	1	1	32
ELOVL FA elongase 1	1	1	78	DAD-1	1	2	32
Nucleoporin Nup160	1	1	77	HSP86	1	1	32
MP68	1	2	75	TIM50	1	1	32
TIMMDC1	1	1	75	Dysferlin	1	1	31
Nrap	1	1	75	COX6C	1	1	30
COMTD1	1	1	75	TRIP-12	1	1	30
IMP-1	1	1	74	Prohibitin	2	2	30
SMIM7	1	1	73	TMEM126A	1	1	29
RPS17	2	2	67	DNA-PKcs	2	2	28
Tim17a	1	1	65	Hp95	1	1	28
EXOC1	1	1	63	UFL1	1	1	28
Sec61 alpha-1	1	1	63	Dnj3	1	1	27
ATP6V0C	1	1	60	MCT 4	1	1	27
SPC22/23	1	1	56	Cytokeratin-10	1	2	26
TMEM209	1	1	55	Beta-III spectrin	1	1	26
N-acetyltransferase 10	1	1	55	ATB(0)	1	1	25
Nudix motif 8	1	1	54	hPAST1	2	2	25
COX7A2	1	1	51	MRM3	1	1	25
Fetuin-A	1	1	50	TMEM111	1	1	24
MTP18	1	1	49	Nucleoporin Nup358	1	1	23
CAS	1	1	49	SCY1-like protein 1	1	1	23
MT-ND2	1	1	48	Alpha1-adaptin	1	1	20
hTom22	1	1	48	SKAP	1	1	20
LMN2R	1	1	48	Alpha-II spectrin	1	1	20
MTA1-L1 protein	1	1	46	DEAD box protein 39	1	1	19
Plectin	2	2	46	SERCA2	1	1	19
Coronin-1C	1	1	44	BiP	1	1	18
MYH-1c	1	1	43	RPS14	1	2	17
NDUFA10	1	1	43	SLC25A12	1	1	16
Ras-like protein TC21	2	2	43	**Sideroflexin-4**	1	1	15
Surfeit locus protein 4	2	2	43	Dystonin	1	1	14
Apoptosis inhibitor 5	1	1	41				

[1] Pept corresponds to the number of distinct peptide sequences.
[2] PSM corresponds to the total number of identified peptide sequences for a protein.

Table S2. Biological processes related to the SFXN1 using PANTHER. Table showing the total number of proteins in each biological process (Group), the number of binding partners identified (Identified), the fold enrichment, the *p* value and the false discovery rate (FDR).

GO biological process complete	Group	Identified	Fold Enrichment	Raw P value	FDR
Mitochondrion organization	438	13	6.44	1.46×10^{-7}	2.55×10^{-4}
Intracellular protein transport	937	18	4.17	$3,01 \times 10^{-7}$	4.29×10^{-4}
Electron transport chain	176	7	8.63	2.17×10^{-5}	1.41×10^{-2}
Establishment of protein localization to organelle	414	10	5.24	2.52×10^{-5}	1.58×10^{-2}
Iron ion homeostasis	83	5	13.07	5.32×10^{-5}	2.77×10^{-2}
L-alpha-amino acid transmembrane transport	44	4	19.72	6.94×10^{-5}	3.51×10^{-2}
Mitochondrial transmembrane transport	89	5	12.19	7.29×10^{-5}	3.57×10^{-2}
Cellular component biogenesis	2666	27	2.20	8.12×10^{-5}	3.74×10^{-2}
Protein import	158	6	8.24	1.10×10^{-4}	4.78×10^{-2}

Table S3. Proteins identified in each biological process. Potential binding partners of SFXN1 with a FDR below 0.05 were analyzed with the online PANTHER classification system using the PANTHER Overrepresentation test (Release date 20171205) and the gene ontology biological process complete data set.

**mitochondrion organization**
Tim17a
Prohibitin
COX7A2
MTP18
TIM50
TIMMDC1
17-beta-HSD 10
hTom22
Prohibitin-2
NDUFA10
HSP86
ATAD3A
MT-ND2

**intracellular protein transport**
RPS14
RPS17
IMP-1
Nrap
Tim17a
Nucleoporin Nup358
DEAD box protein 39
hPAST1
TIM50
Nucleoporin Nup205
Alpha1-adaptin
RAB2B
Nucleoporin Nup160
hTom22
Sec61 alpha-1
Prohibitin-2
SPC22/23
HSP86

**electron transport chain**
SLC25A12
COX6C
COX7A2
COX6A1
COX11
NDUFA10
MT-ND2

**establishment of protein localization to organelle**
RPS14
RPS17
Tim17a
Nucleoporin Nup358
TIM50
hTom22
Sec61 alpha-1
Prohibitin-2
SPC22/23
HSP86

**iron ion homeostasis**
Sideroflexin-1
Sideroflexin-3
Sideroflexin-4
Sideroflexin-2
Sideroflexin-5

**L-alpha-amino acid transmembrane transport**
SLC25A12
CD98
ATB(0)
hLAT1

**mitochondrial transmembrane transport**
Tim17a
SLC25A12
MPC2
TIM50
MFTC

**cellular component biogenesis**
MRM3
RPS14
RPS17
IMP-1
Nrap
hPAST1
CAS
COX7A2
Beta-III spectrin
MYH-1c
N-acetyltransferase 10
TIMMDC1

**cellular component biogenesis**
Nucleoporin Nup205
MGST1
Cytokeratin-10
17-beta-HSD 10
ATB(0)
COX11
Tropomodulin-3
Plectin
Desmoyokin
NDUFA10
Dystonin
GLUT-1
HSP86
Hp95
MT-ND2

**protein import**
Tim17a
Nucleoporin Nup358
TIM50
hTom22
Prohibitin-2
HSP86

Table S4. Biological pathways related to the SFXN1 binding partners using REACTOME. Pathways related to the binding partners of SFXN1 with a FDR below 0.05 using REACTOME. The total number of proteins in each biological process (Group), the number of binding partners identified (Identified), the fold enrichment, the *p* value and the false discovery rate (FDR) are shown.

Biological pathways	Group	Identified	P value	FDR
The citric acid (TCA) cycle and respiratory electron transport	175	9	3.00×10^{-5}	7.27×10^{-3}
Respiratory electron transport	101	7	3.75×10^{-5}	7.27×10^{-3}
Respiratory electron transport, ATP synthesis by chemiosmotic coupling, and heat production by uncoupling proteins.	124	7	1.34×10^{-4}	1.72×10^{-2}
Caspase-mediated cleavage of cytoskeletal proteins	12	3	1.84×10^{-4}	1.79×10^{-2}

Table S5. List of the antibodies used in this study.

Primary antibodies					
**Antibodies**	**Clonality**	**Host Organism**	**Dilution**	**RRID**	**Provider**
Anti-SFXN1	polyclonal	rabbit	1:1000	Sigma-Aldrich Cat# HPA019543, RRID:AB_1856789	Sigma
Anti-SFXN1	polyclonal	rabbit	1:1000	Atlas Antibodies Cat# HPA063745, RRID:AB_2685111	Sigma
Anti-SFXN3	polyclonal	rabbit	1:1000	Atlas Antibodies Cat# HPA048105, RRID:AB_2680265	Sigma
OctA-Probe (H-5) antibody	monoclonal	mouse	1:200	Santa Cruz Biotechnology Cat# sc-166355, RRID:AB_2017593	Santa Cruz
Anti-F1F0 (ATP5b)	monoclonal	mouse	1:1000	Abcam Cat# ab14730, RRID:AB_301438	Abcam
Anti-ATAD3A/B/C	monoclonal	mouse	1:200	Santa Cruz Biotechnology Cat# sc-376185, RRID:AB_10988379	Santa Cruz
Anti-ERAB (17β-HSD10)	monoclonal	mouse	1:200	Santa Cruz Biotechnology Cat# sc-136326, RRID:AB_10647087	Santa Cruz
Anti-Cytochrome C	monoclonal	mouse	1:1000	BD Biosciences Cat# 556432, RRID:AB_396416	BD Biosciences
Anti-TOM40	polyclonal	rabbit	1:200	Santa Cruz Biotechnology Cat# sc-11414, RRID:AB_793274	Santa Cruz
Anti-TOM20	polyclonal	rabbit	1:200	Santa Cruz Biotechnology Cat# sc-11415, RRID:AB_2207533	Santa Cruz
Anti-Tubulin beta	monoclonal	mouse	1:200	Cat# E7, RRID:AB_528499	DSHB
Anti-Tim50	monoclonal	mouse	1:200	Santa Cruz Biotechnology Cat# sc-393678, RRID:AB_2714191	Santa Cruz
Anti-NDUFA10	monoclonal	mouse	1:200	Santa Cruz Biotechnology Cat# sc-376357, RRID:AB_10990305	Santa Cruz
**Secondary antibodies**					
**Antibodies**	**Clonality**	**Host Organism**	**Dilution**	**RRID**	**Provider**
Mouse Normal IgG Control antibody, Unconjugated, Millipore	polyclonal	mouse		Millipore Cat# 12-371, RRID:AB_145840	Millipore
Peroxidase AffiniPure Goat Anti-Rabbit IgG (H+L)	polyclonal	goat	1:5000	Jackson ImmunoResearch Labs Cat# 111-035-003, RRID:AB_2313567	Jackson
anti-mouse HRP-conjugated secondary antibody	polyclonal	goat	1:20,000	Advansta Cat# R-05071-500, RRID:AB_10718209	Advansta
anti-Rabbit AlexaFluor488	polyclonal	goat	1:200	Thermo Fisher Scientific Cat# A-11008, RRID:AB_143165	Thermo Fisher
anti-Rabbit AlexaFluor568	polyclonal	goat	1:200	Thermo Fisher Scientific Cat# A-11011, RRID:AB_143157	Thermo Fisher
anti-Mouse AlexaFluor568	polyclonal	goat	1:200	Thermo Fisher Scientific, catalog # A-11004, RRID AB_2534072	Thermo Fisher

Table S6. Cell lines used in this study.

Cells	Cell Line Description	Provenance	RRID
A2780	Human ovarian carcinoma derived cell line	ECACC, Sigma-Aldrich	ECACC Cat# 93112519, RRID:CVCL_0134
A2780Cis	Cisplatin-resistant human ovarian carcinoma derived cell line	ECACC, Sigma-Aldrich	ECACC Cat# 93112517, RRID:CVCL_1942
COV434	Human ovarian cancer derived cell line	Kind gift from Sandrine Caburet	ECACC Cat# 07071909, RRID:CVCL_2010
HCT116	Human colon cancer derived cell line	Our lab	CVCL_0291
HEK293	Human embryonic kidney 293 cells	Our lab	CVCL_0045
HeLa	Human cervical cancer derived cell line	Our lab	CVCL_0030
HepG2	Human liver carcinoma derived cell line	Kind gift from Delphine Sitterlin	CVCL_0027
HT1080	Fibrosarcoma derived cell line	Our lab	CVCL_0317
KGN	Human granulosa-like tumor derived cell line	Kind gift from Sandrine Caburet	CVCL_0375
MCF-7	Human breast cancer cell line	Kind gift from Philippe Juin	CVCL_0031
MDA-MB-231	Human breast cancer derived cell line	Kind gift from Philippe Juin	CVCL_0062
MDA-MB-468	Human breast cancer derived cell line	Kind gift from Philippe Juin	CVCL_0419
MEFSV40	Mouse immortalized embryonic fibroblasts	Kind gift from Richard Flavell	CVCL_U630
PC12	Rat derived pheochromocytoma	Our lab	CVCL_0481
RKO	Human colon carcinoma derived cell line	Our lab	CVCL_0504
SH-SY5Y	Human neuroblastoma derived cell line	ATCC	ATCC Cat# CRL-2266, RRID:CVCL_0019
T-47D	Human breast cancer derived cell line	Kind gift from Philippe Juin	CVCL_0553

Table S7. Plasmids used in this study (related to Figure S3, purchased from Genscript).

Protein	Cloning Vector	Clone ID	Gene Symbol	Accession Number
SFXN1	pcDNA3.1+/C-(K)-DYK	OHu31628	SFXN1	NM_022754.6
SFXN2	pcDNA3.1+/C-(K)-DYK	OHu14540	SFXN2	NM_178858.5
SFXN4	pcDNA3.1+/C-(K)-DYK	OHu25980	SFXN4	NM_213649.1
SFXN5	pcDNA3.1+/C-(K)-DYK	OHu17388	SFXN5	NM_144579.2
